# Generalized Fano lineshapes reveal exceptional points in photonic molecules

**DOI:** 10.1038/s41467-018-02855-3

**Published:** 2018-01-26

**Authors:** Niccolò Caselli, Francesca Intonti, Federico La China, Francesco Biccari, Francesco Riboli, Annamaria Gerardino, Lianhe Li, Edmund H. Linfield, Francesco Pagliano, Andrea Fiore, Massimo Gurioli

**Affiliations:** 1 European Laboratory for Nonlinear Spectroscopy, via Nello Carrara 1, 50019 Sesto Fiorentino, FI Italy; 2 Department of Physics, University of Florence, via Sansone 1, 50019 Sesto Fiorentino, FI Italy; 3 Instituto de Ciencia de Materiales de Madrid—CSIC, Calle Sor Juana Inés de la Cruz, 3, 28049 Madrid, Spain; 4 Istituto Nazionale di Ottica, CNR, via Nello Carrara 1, 50019 Sesto Fiorentino, Italy; 5 Institute of Photonics and Nanotechnology, CNR, Via Cineto Romano, 42, 00156 Roma, Italy; 6 School of Electronic and Electrical Engineering, University of Leeds, Leeds, LS2 9JT UK; 7 Department of Applied Physics, Institute for Photonic Integration, Eindhoven University of Technology, 5600 MB Eindhoven, The Netherlands

## Abstract

The optical behavior of coupled systems, in which the breaking of parity and time-reversal symmetry occurs, is drawing increasing attention to address the physics of the exceptional point singularity, i.e., when the real and imaginary parts of the normal-mode eigenfrequencies coincide. At this stage, fascinating phenomena are predicted, including electromagnetic-induced transparency and phase transitions. To experimentally observe the exceptional points, the near-field coupling to waveguide proposed so far was proved to work only in peculiar cases. Here, we extend the interference detection scheme, which lies at the heart of the Fano lineshape, by introducing generalized Fano lineshapes as a signature of the exceptional point occurrence in resonant-scattering experiments. We investigate photonic molecules and necklace states in disordered media by means of a near-field hyperspectral mapping. Generalized Fano profiles in material science could extend the characterization of composite nanoresonators, semiconductor nanostructures, and plasmonic and metamaterial devices.

## Abstract (web summary)

Fano lineshapes are found in many photonic systems where discrete and extended spectra interfere. Here, the authors extend this description and introduce generalized Fano lineshapes to describe the results from hyperspectral mapping around an exceptional point in a coupled-cavity system.

## Introduction

Fano profiles were introduced for describing strong asymmetries in the autoionization spectra that arise from the quantum interference between two competing transitions^1–3^. This interference scheme was employed in nuclear, atomic, and solid state physics^4–8^. Later, it was imported into photonics and plasmonics, describing a single or even double resonance systems^9–11^, exploiting the asymmetric lineshape to enhance sensors sensitivity^12^ and using their local changes as an intrinsic interferometer^13,14^. In this picture, Fano profiles are the manifestation of the interference between a resonant mode and a flat background, whose phase difference generates a variety of lineshapes^15^. They are also associated to the quantum electromagnetic-induced transparency (EIT), where the interaction with a metastable state suppresses the resonant absorption, leading to a sharp transmission window^16^. In plasmonics, the coupling of radiative and dark modes can mimic the atomic EIT^17^. In photonic-coupled resonators, a similar sharp transparency window was observed by exploiting destructive interference and by using an adjacent waveguide for measuring the transmission^17–20^. For coupled systems made of two almost resonant building blocks, two different regimes emerge depending on the ratio of the single-loss differences over the mode coupling. The weak-coupling regime (where the normal modes have equal frequencies but different losses) shows a transparency peak-denominated EIT^21^. The second case corresponds to the strong-coupling regime, where the normal modes show different frequencies but identical losses and the spectral response is characterized by the Autler–Townes splitting (ATS), therefore the transparency window is denominated ATS^21^. Notably, at the transition between EIT and ATS exists a singularity, denominated exceptional point (EP), where the two normal states coalesce into a single one. The EP is a feature of open coupled systems related to the breaking of the parity and time-reversal symmetry^21–23^. At the EP, phenomena like quantum-phase transitions and quantum chaos were predicted^24,25^. Among the effects studied in coupled-photonic systems, we mention the transition to a single laser mode^26^, the reversal of the pump dependence in coupled-quantum cascade lasers^27^ and the non-reciprocal wave propagation in coupled waveguides^28^. However, as the EP occurs exactly at the transition between EIT and ATS, it is quite elusive to be detected. The EP transition was revealed by performing coherent measurements, typically observing the transmission through a waveguide (WG) coupled in the near-field to a system of two photonic resonators both in the EIT and ATS regime^21,29^. The major limitation of this scheme is the need for a specific sample that allows a side-coupled WG. Even if it would be possible, accurate conclusions can be drawn only if a large loss-detuning between the two resonators is present and if the WG is coupled only to the lower quality factor cavity. Therefore, a general tool for addressing the close proximity to the EP is actually missing.

Here, we employ resonant scattering (RS) in scanning near-field optical microscopy (SNOM) to reveal EPs. The near-field probe takes the role of the WG and overcomes the limitations explained above. In this scheme, we model the modes interference by introducing the so called generalized Fano lineshapes and link them to the results present in the literature. We investigate a photonic system where the modes of two coupled nanocavities, called photonic molecule, are tailored close to the EP by a post-fabrication control. We observe a wide class of generalized Fano profiles, which nicely agree with the model and with finite-difference time-domain (FDTD) calculations. As a further application, we employ generalized Fano profiles to discriminate individual localized modes versus necklace states in disordered photonics systems .

## Results

### Conceiving experiments close to the exceptional point

**Fig. 1:**
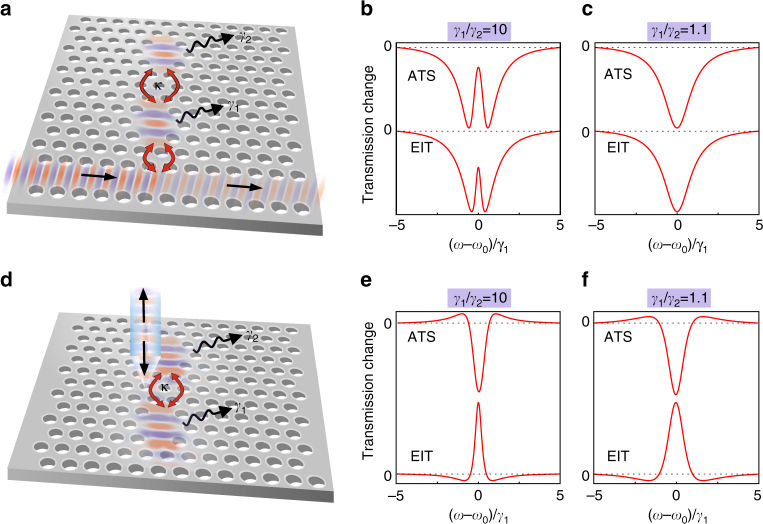
Modeling of waveguide and near-field resonant transmission in a photonic molecule close to the exceptional point. **a** Schematics of two coupled-photonic cavities tested by a waveguide, where the e.m. field is propagating from left to right. The waveguide is coupled (red arrows) to the resonator with losses *γ*_1_, which is also coupled with coupling strength *κ* to the upper resonator with losses *γ*_2_. **b**, **c** Calculated waveguide transmission change due to the presence of the photonic molecule for different values of *κ* to reproduce both the Autler–Townes splitting (ATS) case, for *κ* = 1.2*κ*_0_, and the electromagnetic-induced transparency (EIT) case, for *κ* = 0.8*κ*_0_, with *γ*_1_/*γ*_2_ = 10 and *γ*_1_/*γ*_2_ = 1.1, respectively. The resonant frequencies of the single uncoupled cavities are *ω*_1_ = *ω*_2_ = *ω*_0_. **d** Schematics of the photonic molecule tested by a near-field probe (blue cone) with incoming and outgoing e.m. waves. **e**, **f** Calculated transmission change through the near-field probe for the same cases reported in **b** and **c**, respectively. All spectra are calculated by means of the coupled-mode theory (Supplementary Note 4). Horizontal dashed lines represent the zero value of each lineshape

**Fig. 2:**
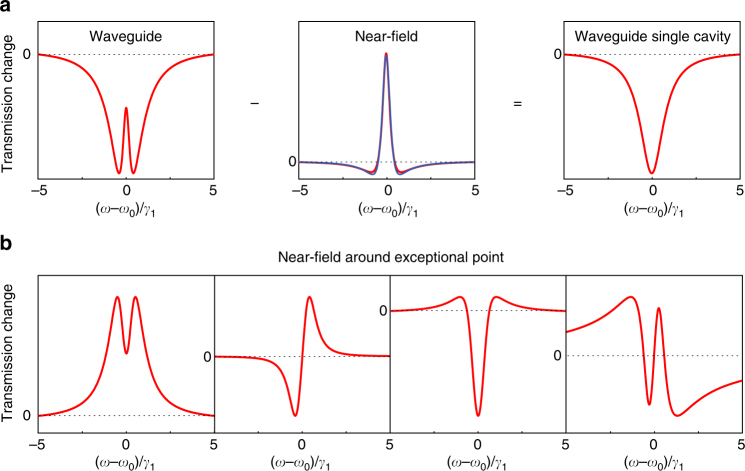
Generalized Fano lineshapes calculation. **a** The difference between the profiles obtained by the waveguide and near-field models, for *κ* = 0.8*κ*_0_ and *γ*_1_/*γ*_2_ = 10, as reported in Fig. 1b and e, gives rise to a Lorentzian dip corresponding to the transmission of the waveguide coupled to a single-cavity system. The near-field generalized Fano profile (central red line) is reproduced by Eq. (1) (blue line) with the fit output parameter *q* = −1. **b** Four representative generalized Fano lineshapes obtained by the near-field model using Supplementary Eq. (61) close to the EP. They show 0, 1, 2, and 3 zero-crossing with the zero-value baseline, respectively

In dielectric-coupled and plasmonic-coupled systems, the mathematical requirements of the EP singularity can be achieved only within the fabrication tolerance. For any unavoidable deviation, the two normal modes show a small frequency splitting (ATS case) or a small-loss difference (EIT case). Our study refers to coupled-photonic resonators that, as built-in initial condition, are characterized by almost degenerate modes in close proximity to the EIT–ATS transition. We designed experiments where the spectral lineshape results from the interference between light confined in coupled modes and light not-resonant with the modes. To refer to the literature, we analyzed the transmission through a waveguide coupled to a photonic molecule, as shown in the schematic of Fig. 1a. Here *κ* is the coupling between the single cavities with losses *γ*_1_, *γ*_2_, and identical frequency (*ω*_1_ = *ω*_2 _= *ω*_0_). The WG is coupled to the cavity with the larger loss (*γ*_1_) only. Spectral lineshapes in the EIT (*κ* < *κ*_0_, where *κ*_0_ = |*γ*_1_ − *γ*_2_|/2) and ATS (*κ* > *κ*_0_) regimes are given for large loss mismatch (*γ*_1_/*γ*_2_ = 10) in Fig. 1b. Although in both cases a sharp transparency window inside a broad Lorentzian dip emerges, the difference is enough to provide a signature of the transition through the EP, by means of the Akaike information criterion^21^. However, the WG approach fails in revealing the EP in many situations. In fact, a clear transparency peak occurs only if *γ*_1_/*γ*_2_ ≳ 3; that is, if the WG is coupled to the cavity with the larger loss and if the initial loss difference is quite large, as highlighted by Fig. 1c, where the peak disappears for *γ*_1_/*γ*_2_ = 1.1. We overcame these limitations by conceiving a resonant-scattering near-field experiment, which allows for a neat discrimination between the two coupling regimes by means of a coherent detection. As reported in the schematic of Fig. 1d, the near-field probe (a tapered optical fiber) replaces the WG, with the advantages of probing both resonators with high-spatial resolution and relaxing all the constraints in the sample design. In a photonic molecule (assuming the tip in a position where it experiences a comparable coupling with both modes), our model predicts lineshapes quite different from standard Fano profiles. In Fig. 1e–f, we calculated the SNOM response using the same parameters of the system tested by the WG. Each of the four spectra (ATS and EIT with *γ*_1_/*γ*_2_ = 10 and 1.1) shows two crossings with its zero-value baseline and an opposite behavior between EIT and ATS profiles. These lineshapes arise from the interference of two resonances with the flat non-resonant scattering, thus showing a physical origin strictly related to standard Fano profiles. Therefore, we denominate them as generalized Fano lineshapes. The detection of generalized Fano lineshapes can reveal the EP independently on the loss mismatch, thus improving the WG approach. The WG and the near-field models are strongly connected around the EP singularity, both for EIT and ATS lineshapes. For instance, the difference between the waveguide transmission and the near-field resonant scattering turns out to be a Lorentzian dip representing the transmission through a WG coupled to a single-cavity system, as shown in Fig. 2a and in Supplementary Note 4. We evaluated the analytical expression of the generalized Fano lineshape, *F*_2_(*ω*), that reproduces the near-field response reported in the central profile of Fig. 2a, see Supplementary Note 3.

1 $$F_2\left( \omega \right) = 2F_0\gamma \frac{{q\left[ {\left( {\omega - \omega _0} \right)^2 - \gamma ^2} \right] + \gamma (q^2 - 1)\left( {\omega - \omega _0} \right)}}{{\left[ {\left( {\omega - \omega _0} \right)^2 + \gamma ^2} \right]^2}}.$$

Here *ω*_0_, *γ* are the resonant frequency and the broadening at the EP and *F*_0_ is an amplitude factor of the mode, whereas *q* is the Fano parameter. Equation (1) is only one example of generalized Fano lineshape. In close proximity to the EP, but by considering a different coupling between photonic modes and near-field tip (i.e., by changing the detection point), different generalized Fano lineshapes showing up to three zero-crossings can be obtained, as reported in Fig. 2b and in Supplementary Notes 3 and 4. All these kinds of profiles are expected when scanning the tip on a coupled system in close proximity to the EP, including the ones observed in the WG configuration. The observation of such profiles proves the occurrence of the EP transition, in the same way that the spectra analysis was exploited in ref. ^21^ Moreover, the near-field method allows for mapping the coupled modes intensities. Last but not least, we were able to relate the profile of *F*_2_(*ω*) to a 2*π* phase jump across the resonance, as well as to identify a further class of analytical expression of generalized Fano profiles with up to three zero-crossings associated to a 3*π* phase jump, whereas the standard Fano profile shows only a *π* phase jump; see Supplementary Note 3.

### Numerical calculations

**Fig. 3:**
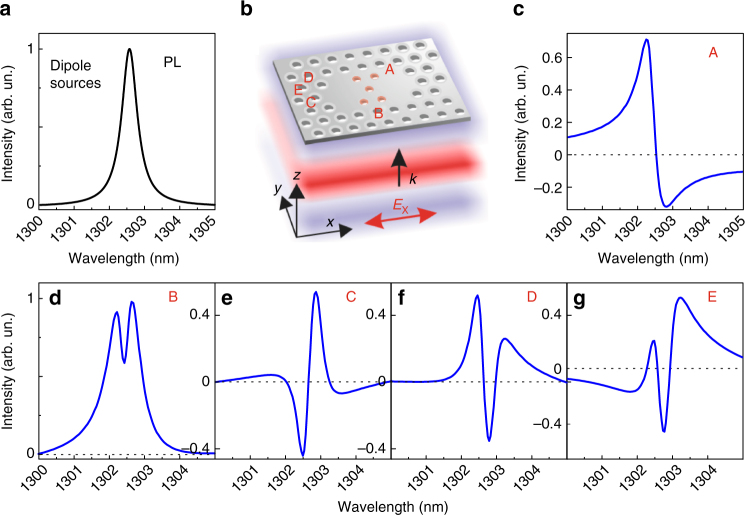
Finite-difference time-domain calculations of near-field resonant scattering in proximity to the exceptional point. **a** The near-field spectrum obtained by employing dipole light sources embedded in the slab, thus resembling the photoluminescence experiment, results in a Lorentzian line. **b** Schematic of the photonic molecule: five central air pores (red circles) are reduced to tailor the coupling. The sketch shows a resonant-scattering simulation where a polarized plane wave is impinging on the slab from the bottom. The plane wave is characterized by the blue and red wavefronts, the wavevector **k** and the electric field polarized along the *x*-direction. A, B, C, D, and E indicate the positions where the spectra reported in **c**–**g** are evaluated. **c**–**g** Near-field spectra of the RS transmission evaluated by employing the layout reported in **b**. The spectrum in **c** is a standard Fano profile. **d**–**g** Spectra exhibiting generalized Fano profiles with 0, 1, 2, 3 crossings with the zero-value baseline, respectively

To study the occurrence of generalized Fano lineshapes in close proximity to the exceptional point, we designed a photonic molecule with small-loss mismatch, of the order of 10%, where the coupling strength is tailored to address the condition (*κ* ~ *κ*_0_; |*ω*_1 _− *ω*_2_| ~ 0). The photonic molecule is composed of two identical nanocavities with modified air pores in the central zone to decrease the coupling^30,31^. FDTD calculations were performed to reproduce the near-field photoluminescence (PL) and resonant-scattering (RS) spectrum, respectively. Hereafter, we will use the wavelength to describe the resonances. Figure 3a shows a typical FDTD spectrum of the nominal system excited by electric dipoles and evaluated on the central position of the photonic molecule. It represents a Lorentzian lineshape and fairly reproduces the PL signal. We found two almost degenerate normal modes exhibiting, between the central position of each cavity, a splitting *λ*_A_ − *λ*_B_ ≈ 0.05 nm (to be compared to *γ*_A_ ~ *γ*_B_ ~ 0.5 nm) and a loss mismatch *γ*_A_/*γ*_B_ = 1.13. These tiny differences suggest that the system is very close to the EP. This also means that by performing only a PL experiment, even in the near-field, it would be challenging to distinguish the case of coupled modes close to the EP with respect to the case of one single mode. To reproduce the resonant-scattering experiment, we performed calculations with the layout reported in Fig. 3b, where the photonic molecule is excited by polarized plane waves. Near-field transmission spectra are calculated in different positions and the notable examples are reported in Fig. 3c–g. A standard Fano profile is found in most positions, as the one in Fig. 3c, since the intensity of one mode dominates over the other, or the phase opposition condition does not occur, see Supplementary Note 3. Where the modes have balanced intensities and a *π* phase difference, a wide variety of generalized Fano profiles up to three crossings with the zero intensity baseline are observed, see Fig. 3d–g. Therefore, numerical calculations prove that it is possible to investigate the EP by the occurrence of generalized Fano lineshapes reproduced by the analytical description in Fig. 2b.

### Experiments on photonic molecule

**Fig. 4:**
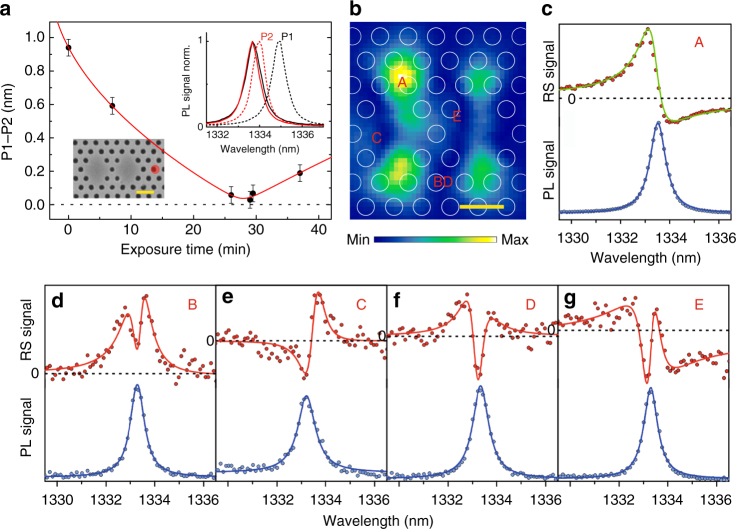
Generalized Fano lineshapes in a photonic molecule. **a** Spectral difference between the modes with larger (P1) and shorter (P2) wavelength as a function of the oxidation-laser exposure time. The red line represents the fit obtained by the coupled-mode theory, see Supplementary Note 8. The error bars are given by the sum of the uncertainties provided by the Lorentzian fit of each mode. Bottom inset: scanning electronic microscope image of the photonic molecule where the red spot marks the position of the oxidation-laser and the scale bar corresponds to 500 nm. Top inset: photoluminescence (PL) spectra collected on the right cavity (where P1 is initially localized) and on the left cavity (where P2 is initially localized) shown as red and black lines, respectively, at *t *= 0 (dashed lines) and at *t *= 29 min (solid lines). **b** Resonant-scattering (RS) amplitude map at *t *= 29 min. A, B, C, D, and E indicate the positions where the spectra of **c**–**g** are collected. **c**–**g** RS and PL data reported as red and blue points, respectively. The blue lines are Lorentzian fits. The green line in **c** is a standard Fano profile fit by Supplementary Eq. (15). The red lines in **d**–**g** are generalized Fano profile fits by Supplementary Eq. (21). Horizontal dashed lines represent the zero value of each RS profile

We fabricated the photonic molecule presented in the previous section on a GaAs membrane, whose scanning electron microscopy (SEM) image is reported in the bottom inset of Fig. 4a. InAs quantum dots in the middle of the slab allow for a straight comparison between PL and RS experiments. By performing near-field PL, we observed two modes (P1 and P2), each localized on a single cavity, split by *Ω* = 0.9 nm with small-loss mismatch (*γ*_P1_/*γ*_P2_ = 1.1, *γ*_P1_ = 0.76 nm, *δγ* = |*γ*_P1_ −* γ*_P2_| = 0.05 nm), see the dashed line spectra in the top inset of Fig. 4a. The observed splitting *Ω* is mainly due to disorder introduced during the fabrication, see Supplementary Note 8. To reduce the initial wavelength detuning, we performed a local laser-oxidation of the membrane^32^, in a position where P1 is more affected than P2, see inset of Fig. 4a, thus producing a selective shift of the mode wavelength. The wavelength difference as a function of the exposure time shows an anticrossing between the two modes, as highlighted in Fig. 4a. The data are nicely reproduced by the coupled-mode theory that exhibits a minimum splitting *Ω*_min_ = (0.03 ± 0.02) nm. This corresponds to the case of zero wavelength detuning, where the relationship ${{\Omega }}_{{\mathrm{min}}}^2 = 4\kappa ^2 - \delta \gamma ^2$ holds. Therefore, the coupling strength is evaluated as 2*κ* = 0.06 nm, see Supplementary Note 8. This proves that at the minimum splitting the investigated photonic molecule is close to the EP (2*κ* = *δγ*) in the ATS regime, even if the coupling is lower than the mode linewidth. The combination of controlling the coupling, having a small-loss mismatch and minimizing the wavelength detuning, allows us to benchmark the existence of generalized Fano profiles in close proximity to the EP. Then, we performed near-field RS at the minimum splitting. The map of the amplitude of the Fano profile^14^ is reported in Fig. 4b. It shows that the modes are delocalized over the entire molecule, even if a larger amplitude is found on the left cavity, likely due to the modification of the right-hand cavity environment by nano-oxidation. In order compare PL and RS spectra acquired in the same point, we report them in Fig. 4c–g for different positions. All the PL spectra are well reproduced by Lorentzian lines. Figure 4c shows spectra acquired where P2 dominates over P1 and in this case the RS is reproduced by a standard Fano profile. On the other hand, the spectra of Fig. 4d–g are collected where the amplitude of the two modes are almost balanced. Here, the RS response is not a standard Fano and shows up to three zero-crossings. These spectra are well reproduced by the developed analytical model for generalized Fano lineshapes, in particular the RS spectrum in Fig. 4d is fitted by Eq. (1). Note that similar lineshapes are found by comparing Fig. 4d–g with Fig. 2b and with the lineshapes predicted by numerical calculation in Fig. 3d–g. Moreover, the spectrum reported in Fig. 4g strongly resembles the reflectivity around the EP found in ref. ^21^ Therefore, a single near-field RS measurement, by showing a generalized Fano profile, proves the proximity to the EP in a coupled system. By exploiting lineshape fitting, we expand the results of ref. ^21^ in monitoring the weak to strong-coupling transition through the EP in cases where the WG approach is not possible. The point is that resonant scattering is an intrinsically interferometric measurement^14^ that exhibits analogies with coherent microscopy and anti-Young interference, in which the Rayleigh limit, either in the spatial domain or in the frequency domain, is overcome^33,34^. We also infer that our approach could detect and quantify small spectral differences (*δλ* and/or *δγ*) between two independent optical signals (if previously individually characterized) as heterodyne mixing would lead to generalized Fano profiles, see Supplementary Note 7.

### Necklace states in disordered photonics

**Fig. 5:**
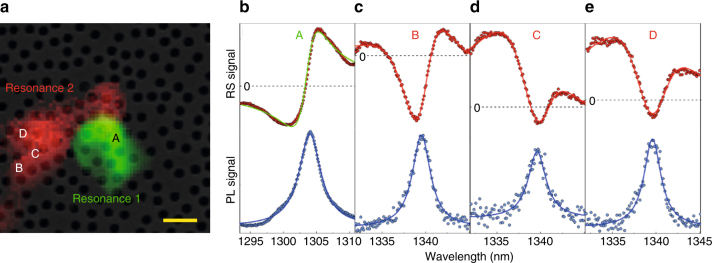
Generalized Fano lineshapes in a disordered photonic medium. **a** Scanning electronic microscope image of the disordered GaAs slab along with the photoluminescence (PL) intensity maps of two spectrally separated resonances superimposed in green and red, respectively. The scale bar is 500 nm. **b**–**e** Comparison between PL and resonant-scattering (RS) spectra detected at the positions A, B, C, D. The PL (RS) data are plotted as blue (red) points. PL data are fitted by Lorentzian (blue lines). **b** The RS spectrum of resonance 1 is reproduced by a standard Fano lineshape (green line). **c**–**e** The RS spectra of resonance 2 are reproduced by generalized Fano profiles (red lines). At every position, the central frequency and the broadening of PL and RS spectra are in agreement

We extended the generalized Fano analysis for retrieving coupled modes around the EP to disordered photonic media. In these systems, the position, the size and the linewidth of resonant modes is almost unknown a priori. Numerical calculations cannot be used to obtain reliable predictions, because additional fluctuations, due to fabrication processes, can drastically change the spectral and spatial distribution of the random modes. At the same time, employing a side-coupled WG is not conceivable. We focus on two-dimensional disordered materials that represent a notable platform for studying mesoscopic effects of photons at low dimensionality^35–38^ and could have a strong impact in the field of renewable energies, telecom and laser applications^39,40^. The local density of optical states in two-dimensional disordered photonic systems can be engineered by tailoring the structural parameters to enhance light localization^41^. Despite the ability to support localized resonances, there exist configurations of the disorder (not predictable a priori) that allow the coupling between adjacent modes, leading to the formation of chains of modes, called necklace states^42^. Their appearance is of utmost importance in determining the light transport. Necklace states have been studied in one-dimensional systems by coherent far-field transmission^43,44^. In photonic systems with larger dimensionality, the role of necklace states has not been studied in detail. In two-dimensional systems, a second-order necklace state has been identified^41^ and, recently, the spatial phase of a given resonance has been proposed for addressing its occurrence^45^. Here, we employ generalized Fano lineshapes for addressing necklace states formed by a collection of coupled random modes, possibly close to the EP (small or zero splitting of the normal modes). Indeed, given the random nature of the modes, which exhibit a large spatial and spectral overlap, we expect to find a considerable number of necklace modes. Moreover, the presented near-field method has the advantage, with respect to any statistical analysis, of being predictive within a single measurement. Evidence of the presence of second-order necklace states would be the simultaneous observation of a Lorentzian in PL and a generalized Fano lineshape in RS. Figure 5a shows the SEM image of the disordered slab with the PL map of two resonances, labeled as 1 and 2, superimposed. The comparison of the PL and RS spectra collected in four different positions are given in Fig. 5b–e. All the PL spectra are reproduced by Lorentzian lines, whose amplitudes define the spatial distributions shown in Fig. 5a, exhibiting a few micrometers localization. By observing the RS spectrum of resonance 1 in Fig. 5b, we infer the presence of a single mode, as it is reproduced by a standard Fano profile. On the other hand, for resonance 2 also generalized Fano profiles are observed, as reported for three different positions in Fig. 5c–e. They show two zero-crossings and are well reproduced by Eq. (1). Then, we conclude that resonance 2 arises from two modes in proximity to the EP and, therefore, it represents a second-order necklace state.

## Discussion

We introduced the family of generalized Fano profiles for describing the coherent optical response of coupled modes in the proximity to the exceptional point singularity. Analytical and numerical predictions were developed and tested at the nanoscale. We demonstrated the ability of the near-field setup, via the detection of generalized Fano lineshapes, to evidence the transition across the exceptional point. The results are a generalization of the waveguide approach, but with improved sensitivity and versatility. We tested the model on ordered photonic molecules and on two-dimensional disordered photonic media. As the Fano formalism provides a correspondence between quantum and classical interference, it could drive the development of novel artificial (meta)materials for quantum photonic applications^46,47^. Our analysis refers to any coupled systems in the proximity of the EP and it could be applicable to excitons in nanostructures and mesoscopic/plasmonic materials^48,49^. New phenomena can be expected in coupled optical resonators close to the exceptional point: the Purcell effect, the strong-coupling of a single photon emitter and a photonic mode, the generation of entangled photon pairs with the same frequency^50,51^. Finally, by revealing the presence of necklace states in disordered media, we believe that generalized Fano profiles will stand as a valuable tool in spectroscopy and material science, not only concerning the optical response but also the electronic properties.

## Methods

### Experimental setup

We used a commercial SNOM (TwinSnom, Omicron GmbH, Taunusstein, Germany) in two different configurations, as highlighted in Supplementary Figure 15. The illumination-collection geometry for both PL and RS employing dielectric near-field probes for investigating PCCs and the transmission geometry employing metal coated near-field probes to detect the RS for disordered system. In PL experiments, the sample is excited with light from a diode laser (780 nm) coupled into a chemically etched optical fiber. In RS experiments, we use light coming from a supercontinuum laser (Leukos-STM, ranging in a photon energy range from 0.8 eV to 1.0 eV, with pulse width of 1 ns and repetition rate of 5 KHz) that is linearly polarized by a Glan-Thompson polarizing prism (extinction ratio 3 × 10^3^). In the illumination-collection geometry, the incoming light is coupled to an optical fiber that ends with a SNOM dielectric tip. A Babinet–Soleil compensator is mounted on the optical fiber to control the light polarization at the end of the tip. The sample is oriented so that the cavity –-axis forms a 45° angle with respect to the input polarization axis. The backward scattered light is collected by the same near-field probe and is filtered in crossed-polarization configuration by a polarizing beam-splitter cube (extinction ratio 2 × 10^4^) to overcome the huge reflection signal, which has the same polarization as that of the incident light. In transmission geometry incoming light is focused on the sample surface by means of a ×50 objective (NA = 0.4) in the far-field regime. The illumination spot diameter is ~2 μm to ensure a homogeneous excitation on the entire PCN plane. Next, the transmitted light is collected by the aluminum-coated probe and the polarizing beam-splitter filters out the polarization components that are orthogonal to the incident light. In both experiments, the signal was collected by the SNOM probe on each desired position of the sample surface, was dispersed by a spectrometer and finally was detected by a liquid nitrogen cooled InGaAs array with a resolution of 0.1 nm. This spectral resolution can be improved by performing Lorentzian lineshape fitting of the data, thus obtaining about 0.02 nm. Regarding the imaging technique, the morphological information given by scanning the near-field probe on the sample surface allows for retrieving the spatial distribution of each spectral line with about 80 nm spatial resolution both in PL and in RS experiments.

### Investigated samples

The PCCs system under consideration is formed of a two-dimensional photonic crystal on a suspended 320-nm-thick GaAs membrane incorporating three layers of high-density InAs quantum dots (QDs) acting as local broad light sources emitting at 1300 nm. The QDs are grown by molecular beam epitaxy at the center of the membrane. The studied structure consists of a two-dimensional triangular lattice of air holes with lattice parameter *a *= 301 nm and air hole diameter size of 193 nm, where each cavity is formed by four missing holes organized in a diamond-like geometry. The system is considered in the so called *K*-coupling, which is formed by two PCCs aligned along the principal *K*-axis of the photonic crystal. Employing a lattice with all identical air pores, strong coupling is expected between the fundamental modes of the single cavities. Starting from the nominal hole diameter of 193 nm, corresponding to a 1.0 nm splitting, we find that the diameter reduction produces a continuous decreasing of the photonic coupling. For a modified hole diameter of 183.2 nm, we find a single resonant mode, meaning that the photonic coupling is decreased down to a value much below the mode broadening. This leads to an accidental degeneracy of the two modes as the C2v symmetry of the *K*-coupled-photonic cavities, cannot support degenerate modes. The planar disordered sample is nanofabricated on GaAs membrane in the same way of the PCCs one. It is characterized by an average hole diameter of 180 nm and an average surface filling fraction of 40%. The strength of scattering in our samples can be quantified by the transport mean-free path ℓ* and in our sample we have kℓ* around 3, therefore indicating strong scattering regime. The samples are optically activated by the inclusion of three layers of InAs QDs at a high number density (10^3^ μm^−2^).

### Finite-difference time-domain calculations

We consider a double cavities system inside photonic crystal on slab with the same nominal parameters of the investigated one. We re-designed the five central air pores to reduce the photonic coupling, following the results of ref. ^18^ The system then shows two accidental-degenerate modes in close proximity to the EP. The first characterization simulates the near-field PL. We employed eight single dipoles emitting in a broad spectral range that covers the system resonances, with both in plane *x* and *y* polarizations. They are placed in the middle of the membrane along the *z*-axis. Although in the *x*–*y* plane, they are placed close to the position where the resonant mode distributions show local maxima, but without any symmetry requirement in the plane of the slab. We detect the emission spectrum at different position in the *x*–*y* plane with small sensors (100 nm × 100 nm) placed at 22 nm on top of the slab surface. We found Lorentzian lineshapes in all points. Between two symmetric points of the photonic molecule localized each on one different cavity, we find a wavelength splitting *δλ* ≈ 0.05 nm (to be compared to *γ*_A _~ *γ*_B _~ 0.5 nm) and a loss mismatch *γ*_A_/*γ*_B_ = 1.1. To simulate near-field transmission, we use plane waves excitation impinging from the bottom of the slab with different angles, ranging from 0° to 25° with respect to the normal to the slab plane, in order to sustain both modes. The plane waves have the same polarization of the resonant modes. Different sensors were placed 22 nm above the sample surface: a big detector covering all the cavities area (2 × 2 μm) that resembles a far-field experiment) and many small (100 × 100 nm) detectors placed in the same area. The former gives a spectrum with a standard Fano profile (very often a Lorentzian one), and no information on the EP presence can be obtained. As the resonant modes have spatial amplitude and phase variations, the amplitude and the phases of the two degenerate modes depend on the position and can rarely be balanced. Then, we expect very different spectra as a function of position. Where the amplitudes of one mode dominates the spectrum is a standard Fano profiles. However, investigating spectra of sensors placed in position where the two modes have comparable amplitudes, we find generalized Fano profiles. Examples are given in Fig. 3d–g and in Supplementary Note 6.

### Data availability

The data supporting the findings of this study are available upon request from the corresponding author (including data presented in the main text and in the Supplementary Information).

## Electronic supplementary material

Supplementary Information
