# Supplementary material for: Generalized Fano lineshapes reveal exceptional points in photonic molecules
Source: Nat Commun. 2018 Jan 26;9:396. doi: 10.1038/s41467-018-02855-3 (PMC5786102; doi:10.1038/s41467-018-02855-3)
Supplement: Supplementary file 1 — Supplementary Information [file 41467_2018_2855_MOESM1_ESM.pdf]

Supplementary Information for:
Generalized Fano lineshapes reveal exceptional points in photonic molecules

Supplementary Note 1: Exceptional point at strong to weak coupling transition

In this section we recall the definition of strong and weak coupling regimes between two coupled cavities, as usually defined in photonics. The system is sketched in Supplementary Figure 1, where A_i is the resonant field in the single cavity, ω_i is the cavity resonance, γ_i is the cavity loss (i =1,2) and κ is the intracavity coupling (here it is a real quantity) given by [1]:

$$\kappa = \frac{2\pi\hbar c}{\lambda}\frac{\int \Delta\varepsilon(\boldsymbol{r})\boldsymbol{E}_0\boldsymbol{r}\cdot\boldsymbol{E}_0(\boldsymbol{r}+\boldsymbol{D})d\boldsymbol{r}}{\int[\mu_0|\boldsymbol{H}_0(\boldsymbol{r})|^2+\varepsilon(\boldsymbol{r})|\boldsymbol{E}_0(\boldsymbol{r})|^2]d\boldsymbol{r}} \tag{1}$$

where $\boldsymbol{E}_0$ and $\boldsymbol{H}_0$ are the electric and magnetic fields of the single cavity; $\boldsymbol{D}$ describes the spatial separation between the cavities; $\Delta\varepsilon$ is the dielectric constant difference between the coupled and the isolated system. Supplementary Equation (1) highlights how the photon tunneling is governed by the spatial overlap of the electric fields, thus depending on the system geometry and on the dielectric permittivity.

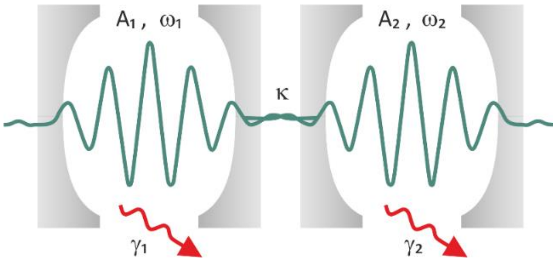

Supplementary Figure 1: Schematics of a photonic molecule. Two optical cavities are coupled by the overlapping coupling parameter κ, given by Supplementary Equation (1). For each single cavity A_i is the resonant field, ω_i is the resonance and γ_i is the loss (i =1,2).

In order to find the normal modes of the coupled system we solve the system:

$$\begin{pmatrix} \omega-\omega_1+i\gamma_1 & -\kappa \\ -\kappa & \omega-\omega_2+i\gamma_2 \end{pmatrix}\begin{pmatrix} A_1 \\ A_2 \end{pmatrix} = \begin{pmatrix} 0 \\ 0 \end{pmatrix} \tag{2}$$

The solutions of Supplementary Equation (2) are normal modes with eigenvalues ω_A, ω_B and linewidths γ_A, γ_B. For sake of simplicity, we deal with the case where the frequency detuning is zero $\omega_1 = \omega_2 \equiv \omega_\mathrm{o}$ and we define $2\gamma = \gamma_1 + \gamma_2$. Therefore the key parameter becomes the loss difference $\delta\gamma = \gamma_1 - \gamma_2$, in fact the normal modes are given by two classes of solutions, distinguished by the condition $2\kappa < |\delta\gamma|$ (weak coupling, WC), and $2\kappa > |\delta\gamma|$ (strong coupling, SC) [2]. The two cases are summarized by:

$$\text{WC:} \quad 2\kappa < |\delta\gamma| \rightarrow \omega_A = \omega_B = \omega_o; \begin{cases} \gamma_A = \gamma + \Gamma \\ \gamma_B = \gamma - \Gamma \end{cases} \text{with } 2\Gamma = \sqrt{\delta\gamma^2 - 4\kappa^2} \tag{3}$$

$$\text{SC:} \quad 2\kappa > |\delta\gamma| \rightarrow \gamma_A = \gamma_B = \gamma; \begin{cases} \omega_A = \omega_o + \Omega \\ \omega_B = \omega_o - \Omega \end{cases} \text{with } 2\Omega = \sqrt{4\kappa^2 - \delta\gamma^2} \tag{4}$$

The WC condition of Supplementary Equation (3) results in normal modes with identical frequency but different linewidths; in SC [Supplementary Equation (4)] the normal modes have different frequencies but identical linewidths. By defining a complex frequency $\widetilde{\omega}_\mathrm{N} = \omega_\mathrm{N} - i\gamma_\mathrm{N}$ (N=A,B) the WC solutions are linked to the SC one by the relationship $\Gamma = i\Omega$. It is worth noting that within this definition SC does not mean that Rabi oscillation will show up, this requires a more stringent condition $\kappa > \gamma$. So, the two normal modes differ either by broadening (WC) or by frequency (SC). However, exactly at the transition, when $2\kappa = |\gamma_1 - \gamma_2|$, the system manifests a singularity that exhibits $\omega_\mathrm{A} = \omega_\mathrm{B}$ and $\gamma_\mathrm{A} = \gamma_\mathrm{B}$, known as exceptional point (EP) [2,3]. By solving the eigenvalues and eigenmodes problem for the coupling matrix in Supplementary Equation (2), we find that it is diagonalized by applying the matrix R and its inverse R^{-1}:

$$R = \begin{pmatrix} 1 & \frac{(2\Omega+i\delta\gamma)}{2\kappa} \\ -\frac{(2\Omega+i\delta\gamma)}{2\kappa} & 1 \end{pmatrix}; \quad R^{-1} = \frac{\kappa^2}{2\Omega^2+i\delta\gamma\Omega}\begin{pmatrix} 1 & -\frac{(2\Omega+i\delta\gamma)}{2\kappa} \\ \frac{(2\Omega+i\delta\gamma)}{2\kappa} & 1 \end{pmatrix} \tag{5}$$

So that Supplementary Equation (2) becomes:

$$\begin{pmatrix} \omega - \omega_o - \Omega + i\gamma & 0 \\ 0 & \omega - \omega_o + \Omega + i\gamma \end{pmatrix} R \begin{pmatrix} A_1 \\ A_2 \end{pmatrix} = \begin{pmatrix} 0 \\ 0 \end{pmatrix} \tag{6}$$

The amplitudes of the normal modes A_A and A_B, with the respect to the uncoupled ones, A_1 and A_2, are given by:

$$\begin{pmatrix} A_\mathrm{A} \\ A_\mathrm{B} \end{pmatrix} = R \begin{pmatrix} A_1 \\ A_2 \end{pmatrix} \quad ; \quad \begin{pmatrix} A_1 \\ A_2 \end{pmatrix} = R^{-1} \begin{pmatrix} A_\mathrm{A} \\ A_\mathrm{B} \end{pmatrix} \tag{7}$$

Note that the amplitudes of A_A and A_B are not-normalized and not-orthogonal, since we are dealing with an open system:

$$\begin{pmatrix} A_\mathrm{A} \\ A_\mathrm{B} \end{pmatrix} = \frac{1}{2\,\kappa} \begin{pmatrix} 2\kappa A_1 + (2\Omega + i\delta\gamma) A_2 \\ -(2\Omega + i\delta\gamma) A_1 + 2\kappa A_2 \end{pmatrix} \xrightarrow{\Omega \ll \delta\gamma} \begin{pmatrix} (A_1 + iA_2) + \frac{2\Omega}{\delta\gamma} A_2 \\ -i(A_1 + iA_2) - \frac{2\Omega}{\delta\gamma} A_1 \end{pmatrix} \tag{8}$$

The last term of Supplementary Equation (8) holds in close proximity of the EP if $\Omega \ll \delta\gamma$, and then for $2\kappa \sim |\delta\gamma|$ (at the first order in Ω). In this case emerges the existence of the EP singularity, where the two normal modes A_A and A_B tend to become the same mode ($A_1 + iA_2$) [3]. The exceptional point is related to anomalous effects in many physical fields from photonics to quantum mechanics [4-6]. Here, we focus on the experimental detection of modes in close proximity to the EP, therefore with almost identical frequency and loss. This occurs when the relation $2\kappa = |\delta\gamma| \neq 0$ is satisfied. The EP singularity does not correspond to the trivial case of two modes with zero-coupling that can be realized by identical cavities largely separated, which do not show interesting physics. We experimentally achieve the condition $2\kappa \sim |\delta\gamma| \neq 0$ for modes exhibiting a large spatial overlap. In close proximity of the EP, in principle we could find either SC regime ($2\kappa > |\delta\gamma|$) with normal modes with same losses γ and a small frequency splitting Ω or WC regime ($2\kappa < |\delta\gamma|$) with normal modes with same frequency ω_o and a small splitting of the losses Γ. Often in the SC case also the condition $\kappa > \gamma$ holds, giving rise to Rabi oscillations as a function of time between the two symmetric and antisymmetric eigenstates. However, in the case where $2\kappa > |\delta\gamma|$ but $\kappa < \gamma$, two different eigenvalues are present, but no Rabi oscillations occur. In photonics no distinction is made between these two regimes [2]. In the field of cavity polariton this circumstance was tentatively defined as critical coupling, where the eigenvalues (frequencies) are split and the modes are mixed, but without Rabi oscillations occurrence [7]; still this definition has not been used by the scientific community and the condition $2\kappa > |\delta\gamma|$ is always defined as strong coupling. In all the presented cases around the transition ($2\kappa \sim |\delta\gamma|$) the solutions are two different modes with almost the same spectral properties, that represent the best achievable approximation of EP. In real coupled systems (e.g. dielectric and plasmonic structures) the mathematical requirements for being exactly at the EP singularity can be achieved only within the fabrication tolerance. Still, they have been attracted great interest, not only in relation to Electromagnetic Induced Transparency (EIT) and Autler Townes splitting (ATS) effects, but also for studying the transition from coupled lasing modes to a single-amplifying laser [4], a reversal of the pump dependence in coupled quantum cascade lasers [8] and the non-reciprocal wave propagation in a coupled two-channel system [9]. In order to detect almost-degenerate modes in close proximity to the EP, we investigate coupled modes found to be in the intermediate coupling regime where both conditions $2\kappa > |\delta\gamma|$ and $\kappa < \gamma_1, \gamma_2$ hold.

Supplementary Note 2: Analytical derivation of standard Fano lineshape with 1 resonance and 1 mode

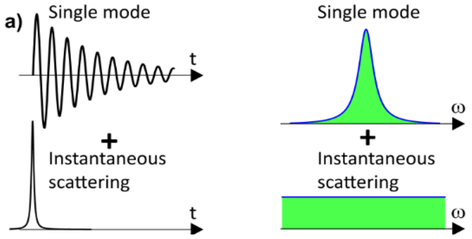

Supplementary Figure 2: Schematics of the interaction between a single resonant mode and a continuum. The single mode is given by a decaying induced dipole. The continuum is given by an instantaneous induced dipole. They are represented both in time and in frequency domains.

Here, we derive the relationship between the semiclassical approach and the standard Fano lineshape. We consider the optical material response as it is due to induced dielectric dipoles, which can be split in two contributions: a non-resonant (instantaneous) response and a resonant (delayed and damped) response. The model schematics is sketched in Supplementary Figure 2 both in the time and frequency domains. Assuming a delta-like temporal excitation (instantaneous scattering) at t =0 and a phase delay φ of the resonant part (single mode), we have:

$$d(t) \propto [c_{\mathrm{NR}}\delta(t) + i\gamma c_{\mathrm{R}} exp\{-\gamma t - i\omega_0 t + i\varphi\}]\vartheta(t) \quad (9)$$

where$c_{\mathrm{NR}}$is the non-resonant amplitude, c_{R} is the resonant amplitude, $\vartheta(t)$ is the Heavyside step function, which accounts for causal response. In the frequency domain, it can be expressed as:

$$d(\omega) \propto c_{\mathrm{NR}} - i\frac{\gamma c_{\mathrm{R}} exp\{+i\varphi\}}{i(\omega-\omega_0)-\gamma} \stackrel{\mathrm{def}}{=} c_{\mathrm{NR}} + i|f_1(\omega)|exp\{i[\theta_1(\omega) + \varphi]\} \quad (10)$$

Here $|f_1(\omega)|^2 = (\gamma c_{\mathrm{R}})^2/[(\omega-\omega_0)^2+\gamma^2]$ is a Lorentzian with resonant frequency ω_o, spectral broadening γ and phase of the resonant dipole equal to $\theta_1(\omega) = arg\{i(\omega-\omega_o)+\gamma\}$ (the amplitude c_{R} is real and positive). From Supplementary Equation (10) we derive the predicted lineshapes for absorption, resonant scattering (RS) and photoluminescence (PL) experiments. First, following [10] we consider absorption measurements, where the total absorption $I_{\mathrm{abs}}(\omega)$ is proportional to the imaginary part of $d(\omega)$. Then we have:

$$I_{\mathrm{abs}}(\omega) \propto Im(c_{\mathrm{NR}}) + |f_1(\omega)|cos\{[\theta_1(\omega) + \varphi]\} \quad (11)$$

The lineshape of the resonant absorption therefore depends on the phase φ. By using a different approach we consider the overall Fano lineshape intensity $Y(\omega)$ given by [11]:

$$Y(\omega) = A + F_0\frac{[\gamma q+(\omega-\omega_0)]^2}{(\omega-\omega_0)^2+\gamma^2} \quad (12)$$

where q is the Fano parameter; $(A+F_0)$ is the background non-resonant signal and therefore the resonant contribution is equal to $[Y(\omega)-(A+F_0)]$. Thus, by mapping the resonant contribution in Supplementary Equation (11) with the resonant contribution in Supplementary Equation (12) we obtain:

$$F_0\gamma\frac{2q(\omega-\omega_0)+\gamma(q^2-1)}{(\omega-\omega_0)^2+\gamma^2} = |f_1(\omega)|cos\{\theta_1(\omega)+\varphi\} \quad (13)$$

that provides the relations $q = -\mathrm{cotan}\{\varphi/2\}$; $F_0 = c_{\mathrm{R}}/(1+q^2)$. Note that we subtracted a flat background with respect to [11]. Supplementary Equation (13) is one main result of [10]. In elastic scattering experiments the signal is proportional to $|d(\omega)|^2$ and in most experiments the non-resonant (instantaneous) scattering is much larger that the resonant (delayed) contribution. Similarly to the absorption, resonant scattering measurements (RS) give Fano profiles, but the relation between q and φ is slightly different. Assuming no absorption from the non-resonant contribution (dielectric material with c_{NR} real) the total elastic scattered signal can be written as:

$$I_{\mathrm{RS}}(\omega) \propto |d(\omega)|^2 \approx |c_{\mathrm{NR}}|^2 + 2c_{\mathrm{NR}}|f_1(\omega)|cos\{\theta_1(\omega)+\varphi+\pi/2\} \quad (14)$$

The scattering signal near ω_0 comes from the interference between the emission from the resonant and non-resonant dipoles. It lies on top of a non-resonant scattering (background) signal due to the instantaneous response of the material. Since elastic scattering is a coherent signal, it brings information on the phase of the optical response. Indeed, it can be either a positive or a negative signal with respect to the background. Thus, mapping the RS lineshape $F_1(\omega)$ after background subtraction with Fano lineshape, we find:

$$F_1(\omega) = F_0\gamma\frac{2q(\omega-\omega_0)+\gamma(q^2-1)}{(\omega-\omega_0)^2+\gamma^2} = 2c_{NR}|f_1(\omega)|cos\{\theta_1(\omega)+\varphi+\pi/2\} \quad (15)$$

Then in resonant scattering measurements the relation between q and φ is:

$$q = -\mathrm{cotan}\left\{\frac{\varphi}{2}+\frac{\pi}{4}\right\} ;\ F_0 = 2c_{\mathrm{NR}}c_{\mathrm{R}}/(1+q^2) \quad (16)$$

The expressions $F_1(\omega,q)$, $\theta_1(\omega)$ and $q(\varphi)$ evaluated in Supplementary Equations (15) and (16) give the lineshapes reported in Supplementary Figure 3. Note that for $\varphi'=\varphi+\pi$ the resonant optical response changes sign. Also the Fano parameters q and F_0 change according to $q' = -1/q$; $F_0{}' = q^2F_0$ both for absorption or scattering. To describe the inversion of the optical signals in the Fano approach, we can use the property that the transformation $(\tilde{q} \to -1/q'$; $\widetilde{F_0} \to -q^2F_0{}')$ does not change the Fano lineshape (i.e. the parameters $q', F_0{}'$ are not unequivocally defined for a given Fano lineshape). It follows that for $\varphi'=\varphi+\pi$, the resulting Fano lineshape can be described by

both the parameter pairs $(q' = -1/q\ ;\ F_0{}' = q^2F_0)$ and $(\tilde{q} = q\ ;\ \widetilde{F_0} = -F_0)$. The latter description has the advantage to highlight the inversion of the Fano lineshape in a straightforward approach. Finally, in PL experiments the signal is proportional to the squared modulus of the resonant part of the dipole:

$$I_{\mathrm{PL}}(\omega) \propto |f_1(\omega)|^2 = \frac{\gamma^2|c_{\mathrm{R}}|^2}{(\omega-\omega_o)^2+\gamma^2} \tag{17}$$

$I_{\mathrm{PL}}(\omega)$ is a Lorentzian lineshape, which does not depend on the phase φ since it is an incoherent signal. In Supplementary Figure 3 a) the phase $\theta_1(\omega)$ and the amplitude $|f_1(\omega)|$ are reported by red and black curves, respectively. Supplementary Figure 3 b) shows the intensity $|f_1(\omega)|^2$ as would be obtained by performing an incoherent measurement, such as PL. Supplementary Figures 3 c)-d) show the interference term $cos[\theta_1(\omega) + \varphi + \pi/2]$ for two typical values of the dephasing with the non-resonant signal $[\varphi = \pi; \varphi = (3/2)\pi]$ and Supplementary Figures 3 e)-f) are the corresponding Fano profiles $F_1(\omega)$ evaluated by Supplementary Equation (15). The phase $\theta_1(\omega)$ jumps by π across ω_o and it is $\theta_1(\omega_o) = 0$. In particular, for $\varphi = \pi$ the interference is constructive (destructive) in the low (high) frequency tail, resulting in the well-known dispersive Fano profile $(q = 1)$ with a zero crossing at $\omega = \omega_o$. On the other hand, for $\varphi = (3/2)\pi$ the term $cos[\theta_1(\omega) + \varphi + \pi/2]$ is always positive and the Fano profile turns out to be a Lorentzian lineshape $(q \gg 1)$ identical to the PL intensity.

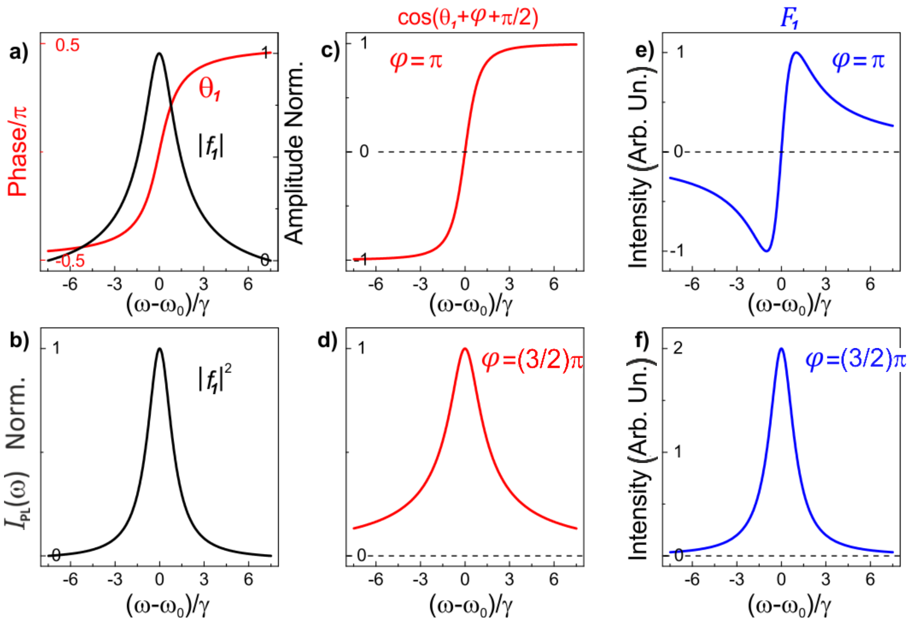

Supplementary Figure 3: Summary of the Fano spectral analysis on a single resonance with one mode. a) Comparison of the amplitude $|f_1(\omega)|$ and phase $\theta_1(\omega)$ as defined in Supplementary Equation (10). b) $|f_1(\omega)|^2$ given by Supplementary Equation (17) that represents a Lorentzian lineshape. c)-d) Interference term $cos\left\{\theta_1(\omega) + \varphi + \frac{\pi}{2}\right\}$ for two notable cases $\varphi = \pi$ and $\varphi = (3/2)\pi$, respectively. e)-f) Scattering amplitude $F_1(\omega)$ given by Supplementary Equation (15) for the two cases of c) and d), respectively. They correspond to Fano profiles with $q = 1$ and $q \gg 1$, respectively.

Supplementary Note 3: Analytical derivation of generalized Fano lineshape with 1 resonance and 2 modes

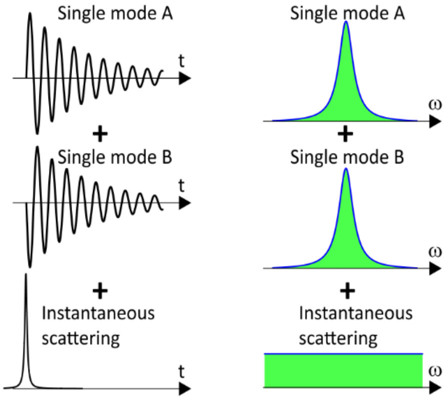

Supplementary Figure 4. Schematics of the interaction between two degenerate and coherent modes with the continuum. The two modes show a phase difference of π between them. The continuum is given by an instantaneous induced dipole. They are represented both in time and in frequency domains.

We derive the $I_{\mathrm{RS}}(\omega)$ lineshape for the case of two degenerate modes A and B, which are the two almost degenerate normal modes in proximity to the EP. We can follow two routes (dipole derivation, Fano derivation) leading to complementary understanding of the physics of resonant scattering in case of one spectral resonance and two modes. In case of two modes, as the ones reported in the schematics of Supplementary Figure 4, the dipole global response in the time domain is:

$$d(t) \propto [c_{\mathrm{NR}}\delta(t) + i\gamma_{\mathrm{A}}c_{\mathrm{A}}exp\{-\gamma_{\mathrm{A}}t - i\omega_{\mathrm{A}}t + i\varphi_{\mathrm{A}}\} + i\gamma_{\mathrm{B}}c_{\mathrm{B}}exp\{-\gamma_{\mathrm{B}}t - i\omega_{\mathrm{B}}t + i\varphi_{\mathrm{B}}\}]\vartheta(t) \quad (18)$$

Where we use the same parameters of the previous derivation. In the frequency domain Supplementary Equation (18) becomes:

$$d(\omega) \propto c_{\mathrm{NR}} - i\gamma_{\mathrm{A}}c_{\mathrm{A}}\frac{exp\{+i\varphi_{\mathrm{A}}\}}{i(\omega-\omega_{\mathrm{A}})-\gamma_{\mathrm{A}}} - i\gamma_{\mathrm{B}}c_{\mathrm{B}}\frac{exp\{+i\varphi_{\mathrm{B}}\}}{i(\omega-\omega_{\mathrm{B}})-\gamma_{\mathrm{B}}} \quad (19)$$

Note, first of all, that by using an incoherent response the signal is given, for any choice of the parameters, by the sum of the modulus square of the two dipoles:

$$I_{\mathrm{PL}}(\omega) \propto \frac{\gamma_{\mathrm{A}}^2|c_{\mathrm{A}}|^2}{(\omega-\omega_{\mathrm{A}})^2+\gamma_{\mathrm{A}}^2} + \frac{\gamma_{\mathrm{B}}^2|c_{\mathrm{B}}|^2}{(\omega-\omega_{\mathrm{B}})^2+\gamma_{\mathrm{B}}^2} \quad (20)$$

which is a sum of two Lorentzian profiles. An interesting behaviour emerges when the spectral properties of the two modes are extremely close. In this case the PL signal gives an overall Lorentzian lineshape from which no information on the relative phase between the two modes can be retrieved. Phase information, which can be obtained in RS experiments, may help in separating two contributions well below the spectral Rayleigh limit. By using Fano derivation we find for the total elastic scattering signal:

$$I_{\mathrm{RS}}(\omega) \approx |c_{\mathrm{NR}}|^2 + F_{1,\mathrm{A}}(\omega) + F_{1,\mathrm{B}}(\omega) =$$

$$= |c_{\mathrm{NR}}|^2 + F_{\mathrm{A}}\gamma_{\mathrm{A}}\frac{2q_{\mathrm{A}}(\omega-\omega_{\mathrm{A}})+\gamma_{\mathrm{A}}(q_{\mathrm{A}}^2-1)}{(\omega-\omega_{\mathrm{A}})^2+\gamma_{\mathrm{A}}^2} + F_{\mathrm{B}}\gamma_{\mathrm{B}}\frac{2q_{\mathrm{B}}(\omega-\omega_{\mathrm{B}})+\gamma_{\mathrm{B}}(q_{\mathrm{B}}^2-1)}{(\omega-\omega_{\mathrm{B}})^2+\gamma_{\mathrm{B}}^2} \quad (21)$$

where the two single Fano resonances $F_{1,\mathrm{A}}(\omega)$ and $F_{1,\mathrm{B}}(\omega)$ are given by Supplementary Equation (15) and related to mode A and B, respectively and q_{A} and q_{B} are linked to φ_{A} and φ_{B} by Supplementary Equation (16), respectively. The resonant scattering results in the sum of two different Fano profiles, which are determined by the interference of mode A and mode B with the non-resonant background, respectively. The point is that, due to the coherent nature of RS, the resulting spectral lineshape can be very different from standard Fano formula, and we define this lineshape as generalized Fano profile. Obviously, in both derivations RS signal for perfect degeneracy ($\omega_{\mathrm{A}} = \omega_{\mathrm{B}}; \gamma_{\mathrm{A}} = \gamma_{\mathrm{B}};\ q_{\mathrm{A}} = q_{\mathrm{B}}$) would be given by a standard Fano profile, as the ones reported in Supplementary Figure 3. However, the perfect degeneracy that is common in quantum systems, is unattainable in photonic materials. In fact, even the astonishing fabrication tolerance at the nanometres length scale cannot guarantee the ideal design, therefore perfect degeneracy is always removed. In addition, even assuming the technological achievement of perfect nanofabrication, the

experimental near-field approach needed for addressing the sufficient spatial resolution, always gives tip induced frequency shifts and additional mode losses, which are unavoidably different for the two modes. Therefore, in photonics resonators a slightly variation from the perfect degeneracy condition always holds and in particular: $\omega_{\mathrm{A}} - \omega_{\mathrm{B}} = \delta\omega$; $\gamma_{\mathrm{A}} - \gamma_{\mathrm{B}} = \delta\gamma$. Here, since the two modes correspond to the normal modes introduced in Supplementary Note 1 we have that $\delta\omega = 2\Omega$. The cases where ($\omega_{\mathrm{A}} \gg \delta\omega$; $\gamma_{\mathrm{A}} \gg \delta\gamma$) can be practically consider as a degeneracy, since in PL measurements Lorentzian lineshapes of mode A and mode B cannot be distinguished. Let us focus on these two cases. We also want to link the signal given in Supplementary Equation (21) with the resonant response as deriving from a single individual effective dipole:

$$d(\omega) \propto c_{NR} + i|f_2(\omega)| exp\{i[\theta_2(\omega) + \varphi_2]\} \tag{22}$$

with $f_2(\omega)$, $\theta_2(\omega)$, φ_2 quantities describing the resonant response, to be derived. We are going to show that this approach is seminal for understanding the link between standard Fano and generalized Fano profile.

Case i): $F_2(\omega)$

Let us consider ($\delta\gamma = 0$; $\gamma = \gamma_{\mathrm{A}} = \gamma_{\mathrm{B}} \gg \delta\omega$) ; in this case (corresponding to close proximity of the EP on the SC side) analytical expressions for the resonant scattering can be derived. We define $\omega_{\mathrm{A}} = \omega_o; \omega_{\mathrm{B}} = \omega_o + \delta\omega > \omega_o$ for a direct comparison with the case of one resonance and one mode. Then in the Fano derivation, we have at the zero-th order in $\delta\omega$.

$$I_{\mathrm{RS}}{}^{0}(\omega) \approx |c_{\mathrm{NR}}|^2 + F_{\mathrm{A}}\gamma \frac{2q_{\mathrm{A}}(\omega-\omega_o)+\gamma(q_{\mathrm{A}}{}^2-1)}{(\omega-\omega_o)^2+\gamma^2} + F_{\mathrm{B}}\gamma \frac{2q_{\mathrm{B}}(\omega-\omega_o)+\gamma(q_{\mathrm{B}}{}^2-1)}{(\omega-\omega_o)^2+\gamma^2} \tag{23}$$

This can be written as a standard Fano profiles in some cases; this is obvious for $F_{\mathrm{A}} = F_{\mathrm{B}}$ and $q_{\mathrm{A}} = q_{\mathrm{B}}$. The interesting case is when $F_{\mathrm{A}} = -F_{\mathrm{B}} = F_0$ and $q_{\mathrm{A}} = q_{\mathrm{B}} = q$, where obviously the resonant part of the zero-th order in $\delta\omega$ vanishes. Therefore, in this case we have to evaluate the first order in $\delta\omega$:

$$I_{\mathrm{RS}}{}^{1}(\omega) = |c_{\mathrm{NR}}|^2 - F_0\gamma \frac{2q(\omega-\omega_o)+\gamma(q^2-1)}{(\omega-\omega_o)^2+\gamma^2} + F_0\gamma \frac{2q(\omega-\omega_o-\delta\omega)+\gamma(q^2-1)}{(\omega-\omega_o-\delta\omega)^2+\gamma^2} \tag{24}$$

$$\mathrm{I}_{\mathrm{RS}}{}^{1}(\omega) \approx |c_{\mathrm{NR}}|^2 + 2F_0\gamma\delta\omega \frac{q[(\omega-\omega_o)^2-\gamma^2]+\gamma(q^2-1)(\omega-\omega_o)}{[(\omega-\omega_o)^2+\gamma^2]^2} \equiv |c_{\mathrm{NR}}|^2 + \delta\omega F_2(\omega) \tag{25}$$

Where $\delta\omega F_2$ is the resonant part of $I_{\mathrm{RS}}{}^{1}(\omega)$, with the definition:

$$F_2(\omega) = 2F_0\gamma \frac{q[(\omega-\omega_o)^2-\gamma^2]+\gamma(q^2-1)(\omega-\omega_o)}{[(\omega-\omega_o)^2+\gamma^2]^2} \tag{26}$$

The coherent response of the system in close proximity to the EP on the SC side, or equivalently of two almost degenerate modes with the same losses, is a peculiar case of generalized Fano lineshapes and can be expressed in a simple analytical formula. In the dipole approach, this particular case can be addressed by defining: $c_{\mathrm{A}} = c_{\mathrm{B}} = c_{\mathrm{R}}$; $\varphi_{\mathrm{B}} = \varphi_{\mathrm{A}} + \pi = \varphi + \pi$ and then we have:

$$d(\omega) \propto c_{\mathrm{NR}} - ic_{\mathrm{R}}\gamma \left[\frac{exp\{+i\varphi\}}{i(\omega-\omega_o)-\gamma} - \frac{exp\{+i\varphi\}}{i(\omega-\omega_o)-\gamma-i\delta\omega}\right] \tag{27}$$

$$\approx c_{\mathrm{NR}} - c_{\mathrm{R}}\gamma\delta\omega \left[\frac{exp\{+i\varphi\}}{(i(\omega-\omega_o)-\gamma)^2}\right] \tag{28}$$

Assuming that the optical response is given by an effective resonant dipole:

$$d(\omega) \stackrel{\mathrm{def}}{=} c_{\mathrm{NR}} + i\delta\omega|f_2(\omega)| exp\{i[\theta_2(\omega) + \varphi]\} \tag{29}$$

Thus, the functions $|f_2(\omega)|$ and $\theta_2(\omega)$ are defined by:

$$|f_2(\omega)| = c_R\gamma[(\omega - \omega_o)^2 + \gamma^2]^{-1} \tag{30}$$

$$\theta_2(\omega) = arg\{i[\gamma^2 - (\omega - \omega_o)^2] - 2\gamma(\omega - \omega_o)\} \tag{31}$$

In Supplementary Figure 5 is shown a summary of these formula. Note that $|f_2(\omega)|$ is a Lorentzian (and not the square root of a Lorentzian, as $|f_1(\omega)|$ is for a single mode) while the resonant phase $\theta_2(\omega)$ jumps by 2π across ω_o, as highlighted by Supplementary Figure 5 a). Following the previous derivation, the scattered signal, subtracted the flat non-resonant contribution, can be written as:

$$F_2(\omega) \propto 2c_{\mathrm{NR}}|f_2(\omega)| cos\{\theta_2(\omega) + \varphi + \pi/2\} \tag{32}$$

Then the 2π jump of $\theta_2(\omega)$ across the resonance leads to anomalous lineshapes for RS. In particular, in Supplementary Figure 5 a) the phase $\theta_2(\omega)$ and the amplitude $|f_2(\omega)|$ are reported by red and black curves, respectively. Supplementary Figure 5 b) shows the PL intensity $PL(\omega) \propto |f_A(\omega)|^2 + |f_B(\omega)|^2$ as it would be obtained by performing any incoherent measurement. Obviously $PL(\omega)$ is a Lorentzian almost identical to the PL signal that would be measured if only the mode A or mode B were present. Supplementary Figures 5 c)-d) show the interference term $cos[\theta_2(\omega) + \varphi + \pi/2]$ for two typical values of the dephasing with the non-resonant signal ($\varphi = \pi, ; \varphi = \pi/2$) and Supplementary Figures 5 e)-f) are the corresponding generalized Fano profiles $F_2(\omega)$. The phase $\theta_2(\omega)$ jumps by 2π across ω_o, therefore for $\varphi = \pi$ the interference character changes two times, resulting in an anomalous Fano profile with two zero crossings. This lineshape is reproduced by equation $F_2(\omega)$ with $q = 1$. To highlight how the lineshape $F_2(\omega)$is given the sum of two single Fano resonances $F_{1,\text{A}}(\omega)$ and $F_{1,\text{B}}(\omega)$ related to mode A and B, respectively, we consider them in Supplementary Figure 5 g) with $q_\text{A} = q_\text{B} = 1$, a small detuning $\delta\omega$, equal linewidth and opposite amplitude. Note that in Supplementary Figure 5 g) we report $F_{1,\text{A}}(\omega)$ and $F_{1,\text{B}}(\omega)$ to highlight the similarity of the two Fano profiles; we also select a quite large detuning to distinguish the two lineshapes. It is clear that, following Supplementary Equation (23), by performing $\left(F_{1,\text{A}} - F_{1,\text{B}}\right)$ we obtain the lineshape of Supplementary Figure 5 e), thus confirming the straightforwardness of our description of anomalous Fano lineshapes. For $\varphi = \pi/2$ the interference character changes sign only one time and the resulting scattering lineshape [Supplementary Figure 5 f)] is a dispersive profile with steeper slopes with respect to the single mode Fano resonance [Supplementary Figure 3 e)]. This lineshape is reproduced by equation $F_2(\omega)$ with $q \gg 1$. As before we consider two single Fano resonances $F_{1,\text{A}}(\omega)$ and $F_{1,\text{B}}(\omega)$ with $q \gg 1$, a small detuning $\delta\omega$, equal linewidth and opposite amplitude. Note that in Supplementary Figure 5 g) we report $F_{1,\text{A}}(\omega)$ and $F_{1,\text{B}}(\omega)$ in order to highlight the similarity of the two Fano profiles and we selected a quite large detuning to distinguish the two lineshapes of Supplementary Figure 5 f). It is clear that, following Supplementary Equation (23), by performing $\left(F_{1,\text{A}} - F_{1,\text{B}}\right)$ we obtain the lineshape of Supplementary Figure 5 f). In all possible cases, note that the expressions of $|f_2(\omega)|$, $\theta_2(\omega)$ and $F_2(\omega)$ do not depend on $\delta\omega$, but its value only affects the amplitude of the RS signal, as highlighted in Supplementary Equation (25).

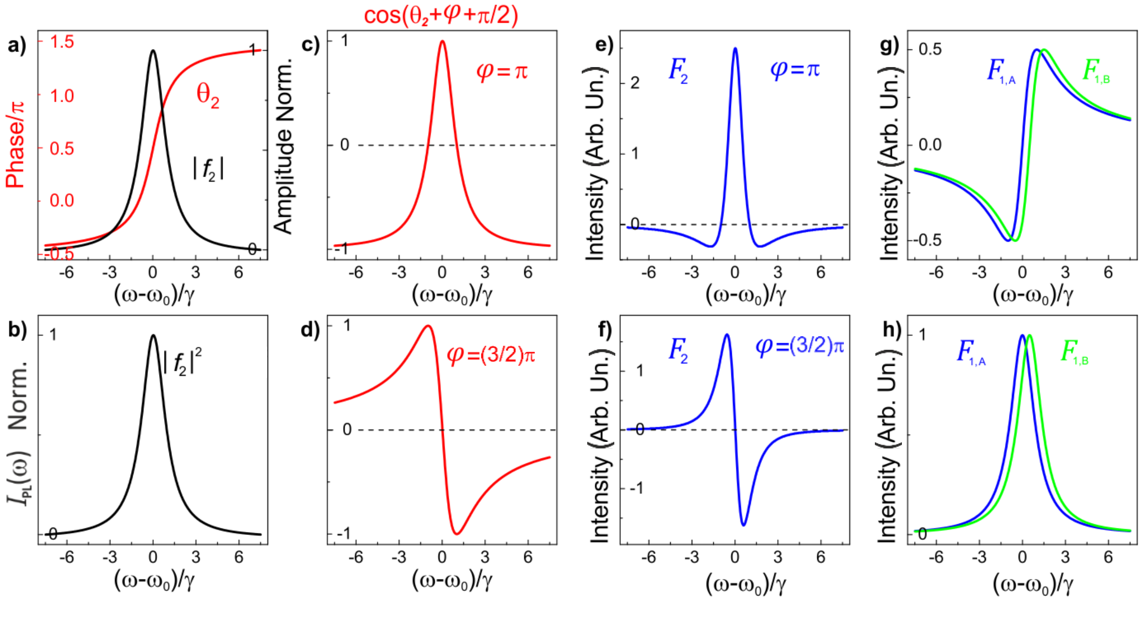

Supplementary Figure 5. Generalized Fano lineshapes for two modes with one resonance and $\delta\omega \ll \gamma$. a) Amplitude $|f_2(\omega)|$ and phase $\theta_2(\omega)$ of the effective dipole. b) $|f_2(\omega)|^2$ given by Supplementary Equation (20) that represents a Lorentzian lineshape. c)-d) Interference term $cos\{[\theta_2(\omega) + \varphi + \pi/2]\}$ for $\varphi = \pi$ and $\varphi = (3/2)\pi$, respectively. e) and f) Scattering amplitude $F_2(\omega)$ for the two cases of c) and d), respectively. g)-h) Standard Fano profiles of the single modes $F_{1,\text{A}}(\omega)$ and $F_{1,\text{B}}(\omega)$, whose subtraction gives the generalized profiles of e) and f), respectively. Here, for a fast comparison we imposed a larger detuning $\delta\omega = \gamma/4$.

Case ii): $F_3(\omega)$

Here we consider the case where $(\delta\omega = 0;\ \omega_A = \omega_B = \omega_o;\ \gamma_B = \gamma_A - \delta\gamma = \gamma - \delta\gamma < \gamma)$, thus corresponding to close proximity of the EP on the WC side. In this case analytical expressions for the resonant scattering can be derived from the Fano analysis. At the zero-th order in $\delta\gamma$ we find:

$$I_{RS}{}^{0}(\omega) \approx |c_{NR}|^2 + F_A\gamma\frac{2q_A(\omega-\omega_o)+\gamma(q_A{}^2-1)}{(\omega-\omega_o)^2+\gamma^2} + F_B\gamma\frac{2q_B(\omega-\omega_o)+\gamma(q_B{}^2-1)}{(\omega-\omega_o)^2+\gamma^2} \quad (33)$$

which can be written as a standard Fano profiles in most cases. As analysed in case *i)*, the interesting case to focus on is $F_A = -F_B = F_0$ and $q_A = q_B = q$, where the zero-th order in $\delta\gamma$ vanishes. At the first order in $\delta\gamma$ we find:

$$I_{RS}{}^{1}(\omega) \approx |c_{NR}|^2 + 2F_0(\omega-\omega_o)\gamma\delta\gamma\frac{q[(\omega-\omega_o)^2-\gamma^2]+\gamma(q^2-1)(\omega-\omega_o)}{[(\omega-\omega_o)^2+\gamma^2]^2} \equiv |c_{NR}|^2 + \delta\gamma F_3(\omega) \quad (34)$$

This is a second peculiar case of generalized Fano lineshape. Note that the shape does not depend on the variation $\delta\gamma$, but only its amplitude. In addition, the resonant part of $I_{RS}{}^{1}(\omega)$, $\delta\gamma F_3$, can be related to the resonant part of $I_{RS}{}^{1}(\omega)$,$\delta\omega F_2$, as:

$$F_3(\omega) = 2F_0\gamma(\omega-\omega_o)\frac{q[(\omega-\omega_o)^2-\gamma^2]+\gamma(q^2-1)(\omega-\omega_o)}{[(\omega-\omega_o)^2+\gamma^2]^2} = (\omega-\omega_o)F_2(\omega) \quad (35)$$

In the dipole approach, case *ii)* is given by choosing: $c_A = c_B = c_R;\ \varphi_B = \varphi_A + \pi = \varphi + \pi$. Then we have:

$$\begin{aligned} d(\omega) \propto c_{NR} - ic_R\left[\frac{\gamma exp\{+i\varphi\}}{i(\omega-\omega_o)-\gamma} - \frac{(\gamma-\delta\gamma)exp\{+i\varphi\}}{i(\omega-\omega_o)-\gamma+\delta\gamma}\right] = \\ = c_{NR} - c_R\delta\gamma\left\{\frac{(\gamma^2-(\omega-\omega_o)^2)+2i\gamma(\omega-\omega_o)}{(\gamma^2+(\omega-\omega_o)^2)^2}\right\}(\omega-\omega_o)exp\{+i\varphi\} \end{aligned} \quad (36)$$

Assuming that the optical response is given by an effective resonant dipole, we have:

$$\begin{aligned} d(\omega) \propto c_{NR} + c_R\delta\gamma\left\{\frac{(\gamma^2-(\omega-\omega_o)^2)+2i\gamma(\omega-\omega_o)}{(\gamma^2+(\omega-\omega_o)^2)^2}\right\}(\omega-\omega_o)exp\{+i\varphi\} = \\ = c_{NR} + i(\omega_o-\omega)\delta\gamma\ f_2(\omega)exp\{+i\varphi\} = \\ \stackrel{\text{def}}{=} c_{NR} + i\delta\gamma|f_3(\omega)|exp\{i[\theta_3(\omega)+\varphi]\} \end{aligned} \quad (37)$$

The right part of this equation defines the functions $|f_3(\omega)|$ and $\theta_3(\omega)$ by:

$$|f_3(\omega)| = c_R\left\{\frac{|(\omega-\omega_o)|}{(\omega-\omega_o)^2+\gamma^2}\right\} \quad (38)$$

$$\theta_3(\omega) = arg\{i[\gamma^2-(\omega-\omega_o)^2] - 2\gamma(\omega-\omega_o)\} + \pi\vartheta(\omega-\omega_o) = \theta_2(\omega) + \pi\vartheta(\omega-\omega_o) \quad (39)$$

Where $\vartheta(x)$ is the Heaviside step function. Note that $|f_3(\omega)|$ is a non-Lorentzian lineshape and the resonant phase $\theta_3(\omega)$ jumps by 3π across the resonance. Indeed a 2π jump is due to the phase of $\theta_2(\omega)$ and an additional discontinuous π jump at $\omega = \omega_o$ is associated to the change of sign of the detuning $(\omega-\omega_o)$. Following the previous derivation, the scattered signal can be written as:

$$I_{RS}{}^{1}(\omega) \approx |c_{NR}|^2 + 2c_{NR}\delta\gamma|f_3(\omega)|cos\left\{\theta_3(\omega) + \varphi + \frac{\pi}{2}\right\} \quad (40)$$

Then the 3π jump of $\theta_3(\omega)$ across ω_o leads to anomalous lineshapes for RS with up to three zeros crossing, as shown in Supplementary Figure 6. In particular, in Supplementary Figure 6 a) the phase $\theta_3(\omega)$ and the amplitude $|f_3(\omega)|$ are reported by red and black curves, respectively. Supplementary Figure 6 b) shows the intensity $I_{PL}(\omega) \propto |f_A(\omega)|^2 + |f_B(\omega)|^2$ as it would be obtained by performing any incoherent measurement. As in case *i)* $I_{PL}(\omega)$ is a Lorentzian almost identical to the PL signal that would be measured if only a single mode was present. Supplementary Figures 6 c)-d) show the term $cos[\theta_3(\omega)+\varphi]$ for two typical values of the dephasing ($\varphi = \pi; \varphi = (3/2)\pi$) and Supplementary Figures 6 e)-f) are the corresponding Fano profiles $F_3(\omega)$. The phase $\theta_3(\omega)$ varies by 3π across the resonance and it is discontinuous by π at ω_o. In general, the interference character changes three times, resulting in an anomalous Fano profile with three zero crossings. This lineshape is reproduced by equation $F_3(\omega)$ with $\varphi = \pi$ (i.e. for $q = 1$). To highlight how the lineshape $F_3(\omega)$ is reproduced by the sum of two single Fano resonances $F_{1,A}(\omega)$ and $F_{1,B}(\omega)$ related to the modes A and B, respectively [as given by Supplementary Equation (21)], we consider them in Supplementary Figure 6 g) with $q_A = q_B = 1$, a small variation $\delta\gamma$, equal amplitude and same ω_o. Note that we choose a quite large $\delta\gamma$ to distinguish the two lineshapes. It is clear that, following Supplementary Equation (33), by performing $F_{1,A}(\omega) - F_{1,B}(\omega)$ we obtain the lineshape of Supplementary Figure 6 e). For $\varphi = (3/2)\pi$ the interference character is always positive and it reaches the zero value only at ω_o. Then the resulting scattering

lineshape [Supplementary Figure 6 f)] is an anomalous profile with a single zero at ω_o. Here, we still consider two single Fano resonances $F_{1,\mathrm{A}}(\omega)$ and $F_{1,\mathrm{B}}(\omega)$ with $q \gg 1$, a small variation $\delta\gamma$, equal ω_o and opposite amplitude. Note that in Supplementary Figure 6 h) we select a quite large $\delta\gamma$ to distinguish the two lineshapes. Finally, note that the expressions of $|f_3(\omega)|$, $\theta_3(\omega)$ and $F_3(\omega)$ do not depend on $\delta\gamma$, but its value only affects the amplitude of the RS signal. Finally, in both Supplementary Equation (32) and (40), the link between q and φ is given by Supplementary Equation (16).

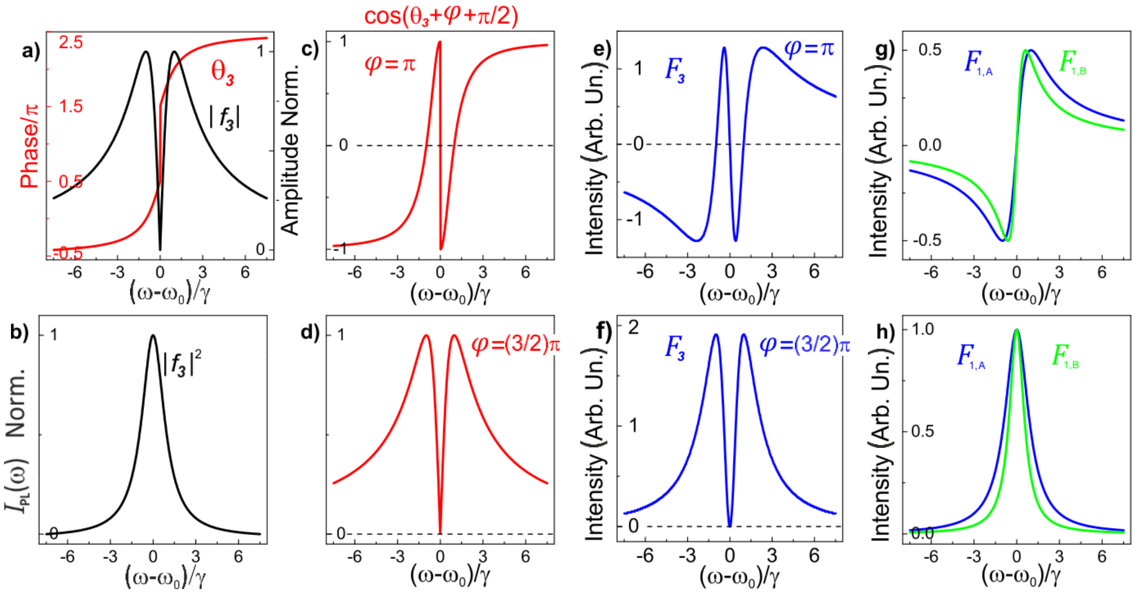

Supplementary Figure 6: Generalized Fano lineshapes for two modes with broadening $\delta\gamma \ll \gamma$.a) Amplitude $|f_3(\omega)|$ and phase $\theta_3(\omega)$ of the effective dipole. b) $|f_3(\omega)|^2$ given by Supplementary Equation (20) that represents a Lorentzian lineshape. c)-d) Interference term $cos\{[\theta_3(\omega) + \varphi + \pi/2]\}$ for $\varphi = \pi$ and $\varphi = (3/2)\pi$, respectively. e)-f) Scattering amplitude $F_3(\omega)$ for the cases of c) and d), respectively. e)-h) Standard Fano profiles for the single modes to be subtracted $(F_\mathrm{A} - F_\mathrm{B})$ to obtain the generalized profiles reported in e) and f). Here, for a fast comparison we imposed a large broadening difference $\delta\gamma = \gamma/4$.

Supplementary Note 4: Probing a coupled system with a waveguide or with a near-field probe

Coupled photonic systems in close proximity to the strong to weak coupling transition have been largely investigated by means of the detection of light transmission through an adjacent waveguide [12-14]. In order to compare our approach with the literature, we compare the spectral response of the resonant scattering through a waveguide (WG) and a near-field probe.

Case i): Single cavity

Here we analyze the case of one single cavity probed by a WG. The system is sketched in Supplementary Figure 7 a).

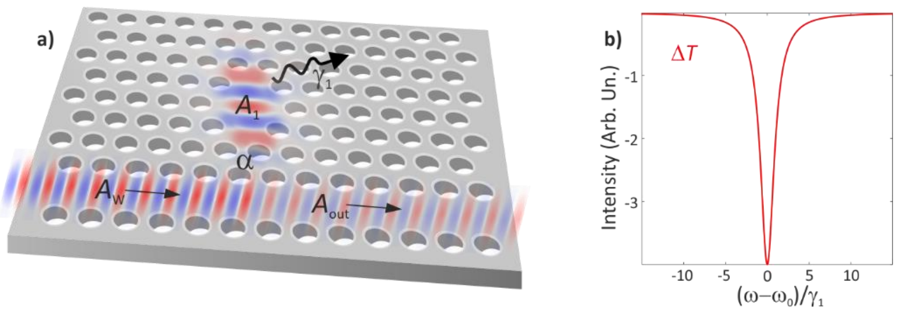

Supplementary Figure 7: Single cavity tested by a waveguide. a) Schematics of the transmission through a waveguide coupled to a single optical cavity. A_W and A_Out describe the fields in the waveguide before and after the interaction with the cavity. $A_1$is the field in the cavity, γ_1 represents the cavity losses, α is the cavity-waveguide coupling. b) Normalized transmission change ΔT, given by Supplementary Equation (46). It shows a Lorentzian dip at the cavity resonance frequency ω_0.

We model the system by following the approach of [2]. In the scalar approximation, the coupled mode theory describes the electric field amplitude that propagates in the WG and that is confined in the cavity by the parameters $A_{\mathrm{W}}$and A_1, respectively [15]. They are defined such that the input power propagating towards the cavity is given by $P_{\mathrm{W}} = |A_{\mathrm{W}}|^2$, the output power propagating in the WG after the cavity results $P_{\mathrm{Out}} = |A_{\mathrm{Out}}|^2$ and that the energy stored in the cavity is $U_{\mathrm{C}} = |A_1|^2$. Note that A_{W} and A_1 are both proportional to the electric fields, but they do not have the same units: $[A_{\mathrm{W}}]/[A_1] = \mathrm{Hz}^{1/2}$. For a stationary pumping we have:

$$(\omega - \omega_o + i(\gamma_1 + \gamma_{\mathrm{W}}))A_1 = -i\alpha A_{\mathrm{W}} \quad (41)$$

$$A_{\mathrm{Out}} = A_{\mathrm{W}} + \alpha A_1 \quad (42)$$

Where ω_o is the cavity resonance, γ_1 is the cavity loss rate outside the WG, γ_W is the cavity loss rate in the WG, α is the WG-cavity coupling, defined as a real quantity with unit $\mathrm{Hz}^{1/2}$. The loss rate γ_{W} is proportional to $|\alpha|^2$; for sake of comparison with EIT models [2] we assume $\gamma_{\mathrm{W}} \ll \gamma_1$. This means that the Q factor of the cavity is not significantly changed by the WG coupling. So A_1 is given by:

$$A_1 = -i\alpha \frac{1}{(\omega - \omega_o + i\gamma_1)} A_{\mathrm{W}} = -i\alpha\chi_1(\omega)\, A_{\mathrm{W}} \quad (43)$$

Where we have defined the frequency-dependent cavity response $\chi_1(\omega)$. By evaluating the field at the end of the WG we get:

$$A_{\mathrm{Out}} = \quad [1 - i\alpha^2\chi_1(\omega)\,]A_{\mathrm{W}} \quad (44)$$

Finally, the transmission (T) normalized to the transmission of the bare WG is:

$$T = \left|\frac{A_{\mathrm{Out}}}{A_{\mathrm{W}}}\right|^2 = |[1 + i\alpha^2\chi_1(\omega)\,]|^2 \cong 1 + 2Im\{\alpha^2\chi_1(\omega)\} \quad (45)$$

where we used the hypothesis of a small WG-cavity coupling: $\alpha^2\chi_1(\omega) \ll 1$ (α is a real quantity). Supplementary Equation (45) coincides with the model used for describing EIT and ATS [3]. In Supplementary Figure 7 b) we report the observable transmission change: $\Delta T = T - 1 = 2Im\{\alpha^2\chi_1(\omega)\}$. The transmission at the end of the WG shows a Lorentzian dip, whose symmetry does not depend on the WG to cavity coupling α, being only an amplitude factor, and, as reported in Supplementary Equation (46), it measures the field in the cavity normalized to the field in the WG.

$$\Delta T = 2Im\left[i\alpha\frac{A_1}{A_{\mathrm{W}}}\right] \quad (46)$$

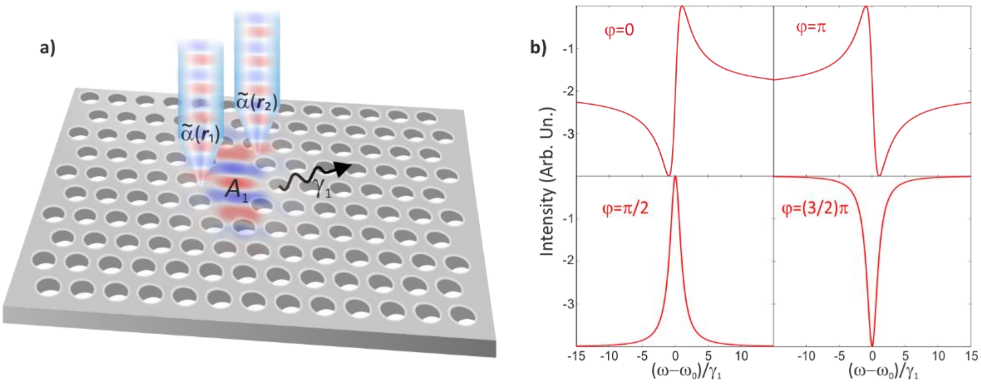

Supplementary Figure 8: Single cavity tested by a near-field probe. a) Schematic of the cavity with resonant field $A_1$and losses γ_1, investigated by a near-field tip (blue cone) in two different positions $\boldsymbol{r}_1$ and $\boldsymbol{r}_2$. $\tilde{\alpha}(\boldsymbol{r})$ is the tip-cavity coupling coefficient. b) Near-field normalized transmission change, given by Supplementary Equation (47), for different phase φ of the coupling term. All these lineshapes are standard Fano profiles.

In the SNOM experiment, sketched in Supplementary Figure 8 a), the single cavity is both pumped and observed in the near-field at the apex of the tip. This approach is denominated resonant back-scattering and it gives Fano lineshapes as the results of the interference between the non-resonant field scattered back by the sample surface and the resonant field in the cavity [16]. Then the resonant scattering intensity is given by the superposition of two fields as in the case of the WG. Indeed, we have shown in [16] that a resonant forward scattering experiment (i.e. a near-field transmission) would give similar results. The equations that describe this model are given in [16] and can be recast in the formalism of the coupled mode theory used in Supplementary Equation (41) and (42). So, A_{w} here represents the field inside the tip and the WG-cavity coupling α is replaced by a tip-cavity position-dependent coupling $\tilde{\alpha}(\boldsymbol{r})$, since the near-field tip can move on the sample surface. Some observed lineshapes are Lorentzian dips

but in general they belong to the wider class of standard Fano profiles [16]. This means that $\tilde{\alpha}$ is a complex quantity, with a phase that is related to the mode phase-distribution. This phase defines the shape of the Fano profiles and the q value or the dipole-phase by Supplementary Equations (16). These lineshapes are reported on Supplementary Figure 8 and they clearly correspond to the standard Fano profiles obtained in Supplementary Note 2. In summary, with respect to the waveguide approach the near-field tip case has the advantage of mapping of the mode phase. The resonant scattering through near-field tip, $I_{\mathrm{RS}}(\omega)$, is given by:

$$I_{\mathrm{RS}}(\omega) \sim 2Im\{\tilde{\alpha}^2\chi_1(\omega)\} \quad \text{where } \tilde{\alpha} = |\tilde{\alpha}|\exp(i\varphi/2) \tag{47}$$

For sake of simplicity, we do not explicit the dependence of $\tilde{\alpha}(\boldsymbol{r})$ on the tip position. If we assume $I_{\mathrm{RS}} \sim 2Re\{\tilde{\alpha}^2\chi_1(\omega)\}$, it would imply a phase shift by $\pi/4$ of the phase φ defined in Supplementary Equation (47). Since we are not investigating the mode-phase retrieval, the conclusion of our analysis would always be that near-field experiments show a variety of Fano profiles as a function of the tip position, in agreement with recent experiments [16].

Case ii): Two coupled cavities

For two coupled cavities the EIT or ATS experiments reported to date are performed by means of a WG coupled only to one single cavity. On the contrary, near-field tip can be positioned either on a single cavity or on the mode overlap region. Supplementary Figure 9 a) shows the sketch of an experiment using WG, which tests, for instance, cavity #1. The system is described by Supplementary Equation (2) with the insertion of the WG field A_{w} and WG-cavity coupling α_1. As in Supplementary Equation (43) we find:

$$\begin{pmatrix} \omega - \omega_1 + i\gamma_1 & -\kappa \\ -\kappa & \omega - \omega_2 + i\gamma_2 \end{pmatrix}\begin{pmatrix} A_1 \\ A_2 \end{pmatrix} = \begin{pmatrix} -i\alpha_1 \\ 0 \end{pmatrix} A_{\mathrm{W}} \quad \rightarrow \quad A_1 = \frac{-i\alpha_1(\omega-\omega_2+i\gamma_2)}{(\omega-\omega_1+i\gamma_1)(\omega-\omega_2+i\gamma_2)-\kappa^2} A_{\mathrm{W}} \tag{48}$$

where κ is the intercavity coupling and $\alpha_1$the coupling between the WG and cavity #1. Here we define the spectral response of the system as:

$$\Upsilon_1(\omega) = \frac{(\omega-\omega_2+i\gamma_2)}{(\omega-\omega_1+i\gamma_1)(\omega-\omega_2+i\gamma_2)-\kappa^2} \tag{49}$$

then, in the limit of small coupling ${\alpha_1}^2$, the normalized transmission change can be written as:

$$\Delta T = 2Im\left[i\alpha_1 \frac{A_1}{A_{\mathrm{w}}}\right] = 2{\alpha_1}^2 Im\{\Upsilon_1\} \tag{50}$$

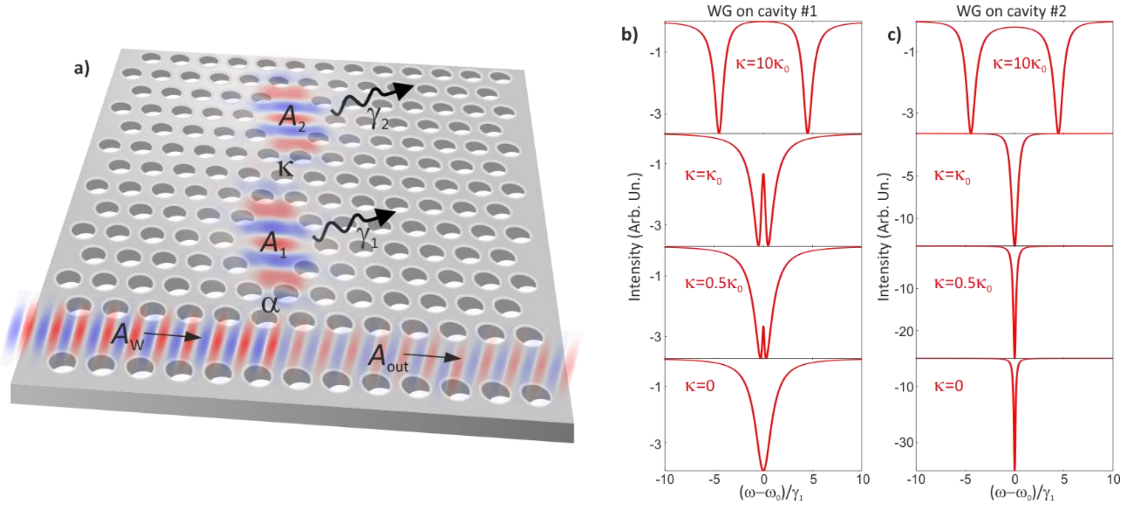

Supplementary Figure 9: Photonic molecule tested by a waveguide coupled to a single cavity. a) Schematic of the photonic molecule where only cavity #1 is coupled to the waveguide (WG) by the coefficient α. A_{W} and A_{Out} describe the fields in the waveguide before and after the interaction with the photonic molecule. A_1 (A_2) and γ_1 (γ_2) are the resonant field and the losses of cavity #1 (cavity #2), respectively. κ is the intercavity coupling. b) Transmission ΔT, as given by Supplementary Equation (50), for the WG coupled to cavity #1 ($\gamma_1 = 10\gamma_2$) for different value of the intercavity coupling κ. At the exceptional point, for $\kappa = \kappa_0 = |\gamma_1 - \gamma_2|/2$, and for $\kappa \sim \kappa_0$ the spectrum shows a clear EIT peak. c) ΔT for the WG coupled to cavity #2. Even at the exceptional point there is no evidence of the EIT signature.

We consider the typical case in which EIT and ATS are reported, that is: $\omega_1 = \omega_2 = \omega_o$, and $\gamma_1 \gg \gamma_2$ [2]. In Supplementary Figure 9 b)-c) we investigate as a function of the coupling κ the normalized transmission change ΔT (for $\gamma_1/\gamma_2 = 10$) as given by Supplementary Equation (50), when the WG is coupled to the cavity #1 and #2,

respectively. Note the striking difference in ΔT when probing the two cavities: the transparency window emerges only by probing the high loss cavity (cavity #1). The coupling κ is varied with respect to the value $\kappa_0 = |\delta\gamma|/2$ that corresponds to the exceptional point (when the normal modes coalesce and $\omega_\mathrm{A} = \omega_\mathrm{B}$, $\gamma_\mathrm{A} = \gamma_\mathrm{B}$). In the strong coupling regime, when $\kappa \gg \kappa_0$, a clear ATS profile with mode frequency splitting with two Lorentzian dips is observed. If the WG is coupled to cavity#1 around the EP transition ($\kappa \sim \kappa_0$), the normal modes tend to coalesce and similar lineshapes, with a sharp transparency window, are observed, as reported in Supplementary Figure 10 a). Still the effect is denominated ATS when it occurs in strong coupling ($\kappa > \kappa_0$) and EIT when in weak coupling ($\kappa < \kappa_0$) regime. For the WG coupled to cavity #1 is we also analyze the lineshapes as a function of the ratio γ_1/γ_2, as shown in Supplementary Figure 10. In each row we used a different coupling strength: $\kappa = 1.2\kappa_0$ (ATS), $\kappa = \kappa_0$ (EP), $\kappa = 0.8\kappa_0$ (EIT), respectively. Clearly for large values of γ_1/γ_2 (around 10) a deep transparency window is always observed, with slight variations between ATS and EIT, that nevertheless can be used to discriminate between the two effects [2]. Moreover, by performing further analysis, in the range $0.9\ \kappa_0 < \kappa < 1.1\ \kappa_0$ the lineshapes tend to be identical, thus meaning that the transition across the EP is smooth. For moderate loss mismatch, $\gamma_1/\gamma_2 < 2$, the transparency window is missing, even at the EP. In summary, the waveguide scheme shows a signature of the EP transition only for large loss mismatch and only if the WG is coupled to the low-Q cavity.

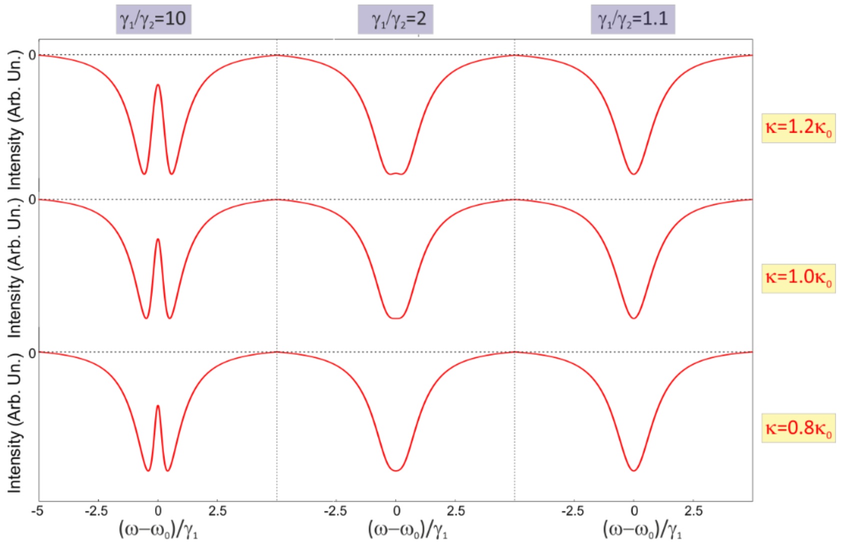

Supplementary Figure 10: Normalized transmission change through a waveguide coupled only to the higher loss cavity of the photonic molecule. ΔT, as given by Supplementary Equation (50) for the waveguide coupled only to cavity #1, as a function of the intercavity coupling κ with respect to $\kappa_0 = |\gamma_1 - \gamma_2|/2$, and as a function of the single cavity loss ratio γ_1/γ_2.

To highlight the meaning of these results, we analytically solve the problem on the normal mode basis recalling Supplementary Note 1, where the transformation matrix R is defined by Supplementary Equation (5):

$$R\begin{pmatrix} \omega - \omega_o + i\gamma_1 & -\kappa \\ -\kappa & \omega - \omega_o + i\gamma_2 \end{pmatrix} R^{-1} R \begin{pmatrix} A_1 \\ A_2 \end{pmatrix} = R \begin{pmatrix} -i\alpha_1 \\ 0 \end{pmatrix} A_\mathrm{W} \tag{51}$$

This leads to:

$$\begin{pmatrix} \omega - \omega_o - \Omega + i\gamma & 0 \\ 0 & \omega - \omega_o + \Omega + i\gamma \end{pmatrix} \begin{pmatrix} A_\mathrm{A} \\ A_\mathrm{B} \end{pmatrix} = \begin{pmatrix} -i\alpha_\mathrm{A} \\ -i\alpha_\mathrm{B} \end{pmatrix} A_\mathrm{W} \tag{52}$$

Supplementary Equation (52) applies both to SC regime, where $2\Omega = \sqrt{4\kappa^2 - \delta\gamma^2}$ is a real quantity, and to WC regime where $2\Omega = -i\Gamma = -i\sqrt{\delta\gamma^2 - 4\kappa^2}$ is a pure imaginary quantity. Then the normal eigenvectors result:

$$\begin{pmatrix} A_\mathrm{A} \\ A_\mathrm{B} \end{pmatrix} = \begin{pmatrix} -i\alpha_\mathrm{A} \frac{1}{\omega - \omega_o - \Omega + i\gamma} \\ -i\alpha_\mathrm{B} \frac{1}{\omega - \omega_o + \Omega + i\gamma} \end{pmatrix} A_\mathrm{W} \equiv \begin{pmatrix} -i\alpha_\mathrm{A}\chi_\mathrm{A}(\omega) \\ -i\alpha_\mathrm{B}\chi_\mathrm{B}(\omega) \end{pmatrix} A_\mathrm{W} \tag{53}$$

By pumping cavity #1 we excite both normal modes with different couplings given by:

$$\begin{pmatrix} \alpha_A \\ \alpha_B \end{pmatrix} = \frac{\alpha_1}{2\,\kappa}\begin{pmatrix} 2\,\kappa \\ -(2\Omega + i\delta\gamma) \end{pmatrix} \tag{54}$$

and each normal mode has a Lorentzian response, $\chi_A(\omega)$ and $\chi_B(\omega)$, with a single pole. Then, since the WG coupled to cavity #1, to get the WG transmission we evaluate A_1:

$$A_1 = -i\frac{2\kappa^2}{4\Omega^2+2i\delta\gamma\Omega}\left(\alpha_A\chi_A - \frac{2\Omega+i\delta\gamma}{2\,\kappa}\alpha_B\chi_B\right)A_W \tag{55}$$

or:

$$A_1 = -i\alpha_1\frac{\kappa}{2\Omega}\left(\frac{(2\Omega-i\delta\gamma)}{2\,\kappa}\chi_A + \frac{2\Omega+i\delta\gamma}{2\,\kappa}\chi_B\right)A_W \tag{56}$$

Then, given Supplementary Equation (50) $\Delta T = 2Im[i\alpha_1 A_1/A_W]$, both EIT and ATS arise from the sum of the response of the two normal modes. In ATS for a large frequency splitting ($\Omega \cong 2\,\kappa \gg \delta\gamma$) the field $A_1$is given by the in phase contribution response of the two normal modes:

$$A_1 \cong -i\alpha_1\frac{1}{2}(\chi_A + \chi_B)A_W \tag{57}$$

Approaching the degeneration, that is close to the exceptional point ($2\,\kappa \cong \delta\gamma\,, \Omega \cong 0$), but still in strong coupling, we have:

$$A_1 = -i\frac{\alpha_1}{2}\left((\chi_A + \chi_B) + i\frac{\delta\gamma}{2\Omega}(\chi_B - \chi_A)\right)A_W \tag{58}$$

In weak coupling A_1 is the same as Supplementary Equation (58) with the substitution Ω=-$i\Gamma$. The term $(\chi_B - \chi_A)$ in Supplementary Equation (58) comes from the destructive interference between the responses of the normal modes and it corresponds to a generalized Fano lineshape. We conclude that near the EP the transparency window is a generalized Fano response, which is the signature of proximity to degeneration. This is highlighted in Supplementary Figure 11 where the two contributions of Supplementary Equation (58) are plotted independently. The central panel reports $\frac{\delta\gamma}{2\Omega}(\chi_B - \chi_A)$ and it also represents the generalized Fano lineshape F_2 described in Supplementary Equation (26). Note that if $\delta\gamma < 0$ (corresponding to the case of WG coupled to high Q cavity, that is cavity #2 in our scheme) the F_2 lineshape is reversed and the transparency window disappears. This explains the results in Supplementary Figure 9 c): when probing cavity #2 the signature of mode degeneracy F_2 is hidden by the presence of the first tem of Supplementary Equation (58), i.e. a Lorentzian. Similarly, for the case of $|\delta\gamma| \ll \gamma$ the amplitude of the generalized Fano F_2 is strongly reduced and then mode degeneracy cannot be detected with the WG approach. Finally, at the exceptional point ($2\,\kappa = \delta\gamma\,, \Omega = 0$) the decomposition in normal modes fails, while Supplementary Equation (50) is still valid, thus showing that the response function has a pole of second order in addition to the usual pole of first order [3]. We thus conclude that the generalized Fano term F_2 in Supplementary Equation (58) corresponds to the part of the EP response with a second order pole.

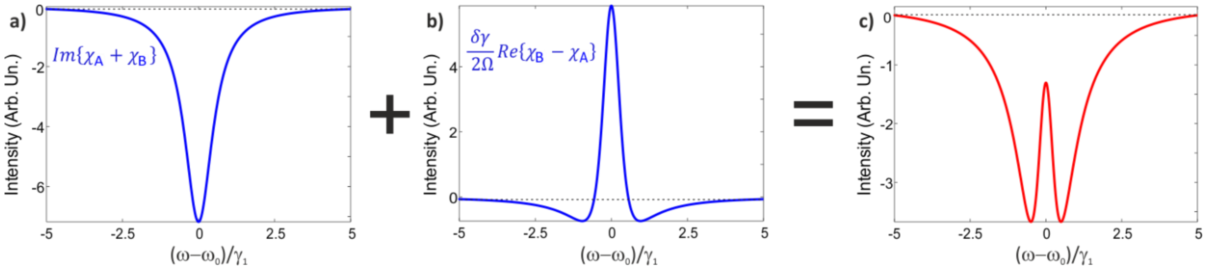

Supplementary Figure 11: Graphic interpretation of Supplementary Equation (58). a)-b) The quantity $Im\{\chi_A + \chi_B\}$ and $\frac{\delta\gamma}{2\Omega}Re\{\chi_B - \chi_A\}$ are evaluated, respectively, in the strong coupling regime but close to the EP singularity. c) The sum of the blue curves in a) and b) results in a generalized Fano lineshape (red line). The parameters used are: $\kappa = 1.01\kappa_0$; $\gamma_1/\gamma_2 = 10$; $\alpha_1 = 2$.

In near-field experiments the tip can be coupled, with a complex term, to both cavities. The corresponding equations (still imposing $\omega_1 = \omega_2 = \omega_o$) are:

$$\begin{cases}(\omega-\omega_0+i\gamma_1)A_1-\kappa A_2=-i\tilde{\alpha}_1 A_{\mathrm{W}}\\(\omega-\omega_0+i\gamma_2)A_2-\kappa A_1=-i\tilde{\alpha}_2 A_{\mathrm{W}}\end{cases}\quad\rightarrow\quad\begin{cases}A_1=-i\,\frac{\tilde{\alpha}_1(\omega-\omega_0+i\gamma_2)+\tilde{\alpha}_2\kappa}{(\omega-\omega_0+i\gamma_1)(\omega-\omega_0+i\gamma_2)-\kappa^2}A_{\mathrm{W}}\\A_2=-\,i\,\frac{\tilde{\alpha}_2(\omega-\omega_0+i\gamma_1)+\tilde{\alpha}_1\kappa}{(\omega-\omega_0+i\gamma_1)(\omega-\omega_0+i\gamma_2)-\kappa^2}A_{\mathrm{W}}\end{cases}\qquad(59)$$

Where $\tilde{\alpha}_2$ gives the coupling between the tip and cavity #2. By changing the tip position we can vary the ratio $\tilde{\alpha}_1/\tilde{\alpha}_2$. Then by defining:

$$\widetilde{Y_1}(\omega)=\frac{(\omega-\omega_2+i\gamma_2)+\tilde{\alpha}_2\kappa/\tilde{\alpha}_1}{(\omega-\omega_1+i\gamma_1)(\omega-\omega_2+i\gamma_2)-\kappa^2};\qquad \widetilde{Y_2}(\omega)=\frac{(\omega-\omega_1+i\gamma_1)+\tilde{\alpha}_1\kappa/\tilde{\alpha}_2}{(\omega-\omega_1+i\gamma_1)(\omega-\omega_2+i\gamma_2)-\kappa^2}\qquad(60)$$

the detected resonant change is a sum of two Fano profiles, each given by Supplementary Equation (46), thus the transmission through the near-field probe $I_{\mathrm{RS}}(\omega)$ results:

$$I_{\mathrm{RS}}(\omega)\ \sim 2Im\left(\tilde{\alpha}_1{}^2\widetilde{Y_1}\right)+2Im\left(\tilde{\alpha}_2{}^2\widetilde{Y_2}\right)\qquad(61)$$

where $\tilde{\alpha}_1=|\tilde{\alpha}_1|\exp(i\varphi_1/2)$, $\tilde{\alpha}_2=|\tilde{\alpha}_2|\exp(i\varphi_2/2)$. In order to make a comparison with the case where the WG probes cavity #1, we assume $\tilde{\alpha}_2=0$. The transmission change through a near-field tip in different positions on a photonic molecule as a function of the coupling strength are shown in Supplementary Figure 12, for the same parameters used in Supplementary Figure 9. When $\varphi_1=0$ the SNOM lineshape coincides with the WG transmission, that is with the imaginary part of the system response $\widetilde{Y_1}$. However, by selecting $\varphi_1=\pi/2$ the lineshapes become dispersive, and near the EP (both in EIT or ATS) the SNOM approach offers the possibility to measure also the real part of the response $\widetilde{Y_1}$, which in the WG approach is related to the slow light effect [12,17]. In summary, the same results of the WG setup, both in EIT and in ATS, can be retrieved by the SNOM approach, which also gives access to the real part of $\widetilde{Y_1}$.

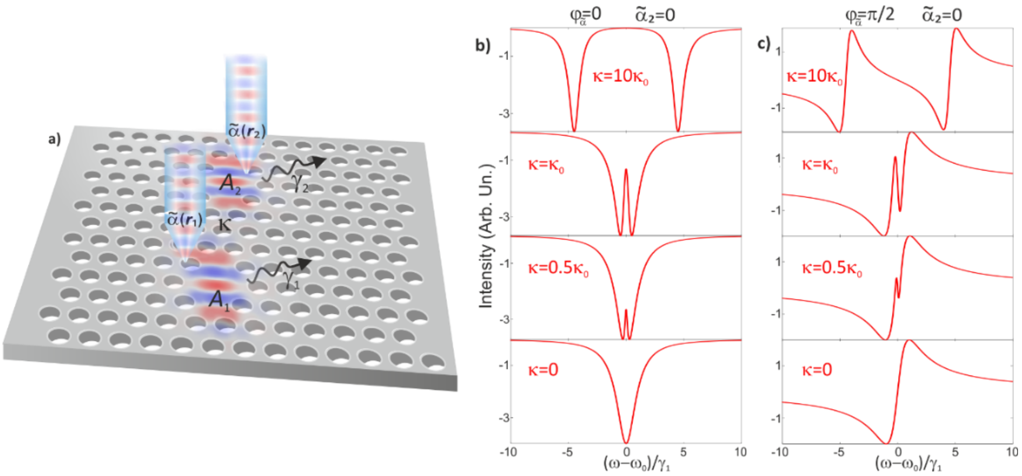

Supplementary Figure 12: Photonic molecule tested by a near-field probe in different position as a function of the coupling strength. a) Schematic of the photonic molecule investigated by a near-field tip (blue cone) in two positions $\boldsymbol{r}_1$ and $\boldsymbol{r}_2$. $\tilde{\alpha}(\boldsymbol{r})$ is the tip-cavity coupling coefficient. A_1 (A_2) and γ_1 (γ_2) are the resonant field and the losses of cavity #1 (cavity #2), respectively. κ is the intercavity coupling. b) Tip transmission change given by Supplementary Equation (61) with $\varphi_1=0$ for different value of κ. c) Tip transmission change calculated for $\varphi_1=\pi/2$. This shows that near-field approach can also detect the real part of the system response. The parameters used for the calculations are $\gamma_1/\gamma_2=10$ and $\tilde{\alpha}_2=0$.

The main novelty of the near-field approach is the detection of generalized Fano lineshapes near the EP for any values of γ_1/γ_2. By exploiting the collection from both cavities, we can cancel the two standard Fano contributions in Supplementary Equation (58), by balancing the collection ($\tilde{\alpha}_1{}^2=-\tilde{\alpha}_2{}^2$). In order to compare the results to the WG approach, in Supplementary Figure 13 we show the calculation performed using Supplementary Equation (61). The generalized Fano lineshape F_2, fingerprint of the proximity to the EP, is always observed. Approaching the EP, the lineshape does not change, but its amplitude tends to zero. Exactly at the EP we get zero signal due to complete destructive interference. Still, for any small deviation from the EP (or even for $\tilde{\alpha}_1{}^2\neq-\tilde{\alpha}_2{}^2$) the SNOM approach furnishes generalized Fano lineshapes, as reported on Supplementary Figure 14. In conclusion, our method is a generalization of the WG coupling commonly used for probing EIT or ATS transmission peaks. The near-field approach retrieves the same results when probing only one single cavity but possess the noteworthy advantage to explore also the real part of the system response, related to the group velocity effect.

Moreover, the transition between ATS and EIT can be distinguishable by a sudden change of the generalized Fano lineshapes observed in resonant scattering as a function of the inter-cavity coupling, thus extending the possibilities of the WG approach.

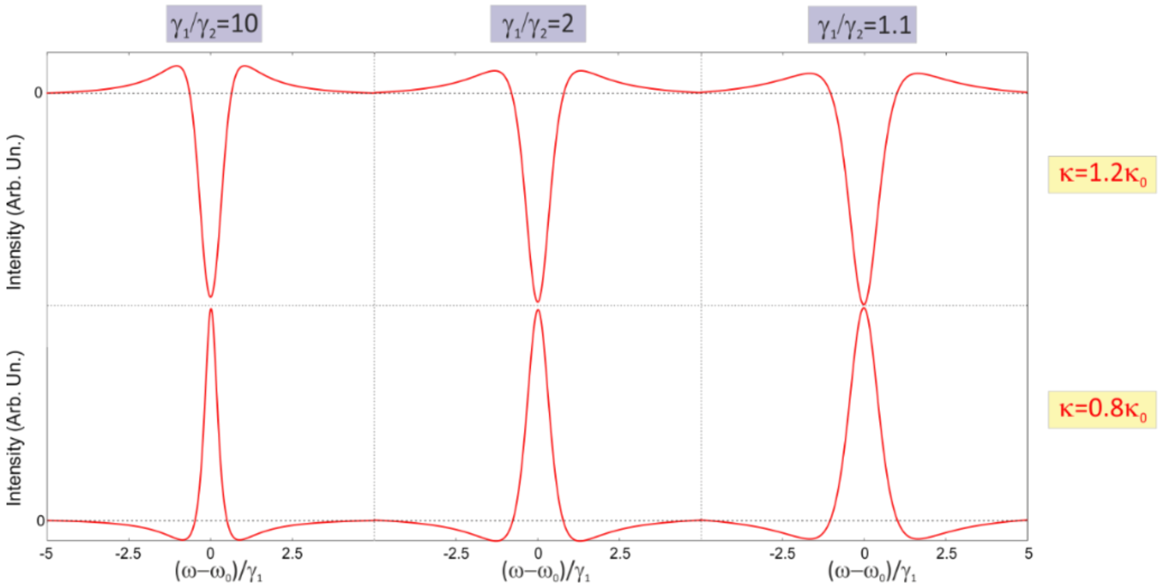

Supplementary Figure 13: Calculation performed using Supplementary Equation (61) near the exceptional point. The near-field detection is evaluated both in strong coupling (κ=1.2 κ_0) and weak coupling (κ=0.8 κ_0) conditions, as a function of the single cavity loss ratio γ_1/γ_2.and for the pumping condition of $\tilde{\alpha}_1{}^2 = -\tilde{\alpha}_2{}^2$.

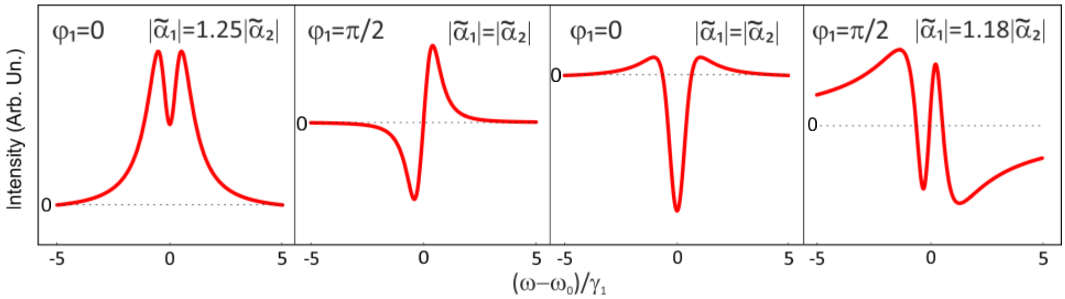

Supplementary Figure 14: Generalized Fano lineshape calculated employed Supplementary Equation (61). They are four notable spectra obtained by the near-field model. For every spectrum the calculation parameters are $\gamma_1/\gamma_2 = 10$, $\omega_1 = \omega_2$, κ=1.2 κ_0. While we changed the phase difference φ_1 with the non-resonant background, as well as the coupling amplitude between the near-field probe and the two cavities $|\tilde{\alpha}_1|/|\tilde{\alpha}_2|$.

Supplementary Note 5: Experimental setup

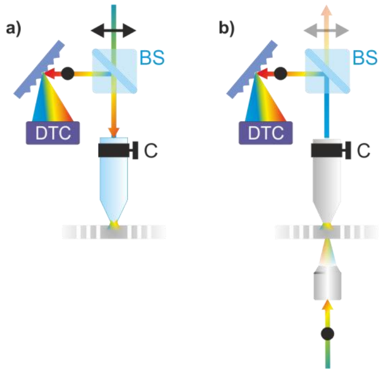

Supplementary Figure 15. Experimental configurations for resonant scattering experiments. a) Schematics of the SNOM setup in illumination-collection geometry. Light from a supercontinuum laser transmitted through a polarizing beam-splitter cube (BS) is linearly polarized and it is coupled to the SNOM dielectric tip. The backward scattered light collected by the probe is filtered in crossed-polarization configuration by the BS, dispersed by a spectrometer and finally detected by a cooled InGaAs array (DTC). b) Schematics of the SNOM setup in transmission geometry The supercontinuum laser light, passes through a linear polarizer film and then illuminates the bottom of the sample by means of a 50X-objective (NA=0.4). The same polarization component of the forward scattered light that is collected by the aluminium -coated aperture probe is reflected by the BS to the detection unit. In both geometries, a Babinet–Soleil polarization compensator (C) is mounted on the optical fibre.

Supplementary Note 6: Further examples of generalized Fano lineshape

Here, we discuss more generally the possible lineshapes for one and two resonances. Supplementary Note 2 deals with two analytical examples of anomalous Fano lineshapes when $F_{\mathrm{A}} = -F_{\mathrm{B}}$, $q_{\mathrm{A}} = q_{\mathrm{B}}$ and either $\delta\omega \neq 0$ or $\delta\gamma \neq 0$, respectively. A much larger variety of generalized Fano lineshapes can be delivered assuming small amplitude variations $F_{\mathrm{A}} = -F_{\mathrm{B}} + \delta F$ and/or Fano parameter $q_{\mathrm{A}} = q_{\mathrm{B}} + \delta q$, in addition to $\delta\omega$ and/or $\delta\gamma$. In these cases, the problem must be handles numerically. In the main text the near-field experimental data of the photonic molecule reported Fig. 4 are fitted by Supplementary Equation (21). In Supplementary Table 1 we show the fitting output parameters. Note that the central frequency and broadening in the different fits slightly depends on the detection points. This is due to the tip perturbation effect as discussed in [16].

Position	ω_{A} (nm)	ω_{B} (nm)	γ_{A} (nm)	γ_{B} (nm)	q_{A}	q_{B}	$F_{\mathrm{A}}/F_{\mathrm{B}}$
B	1333.22	1333.42	0.49	0.29	0.38	0.88	-1.6
C	1333.43	1333.59	0.50	0.35	35	35	-0.82
D	1333.15	1333.36	0.39	0.41	-1.27	-0.99	-0.84
E	1333.28	1333.29	0.45	0.37	1.0	1.0	-0.99

Supplementary Table 1: Output parameters of the fitting with Supplementary Equation (21) of the experimental near-field generalized Fano lineshapes observed in the photonic molecule reported on the main text. The position B, C, D, and E correspond to the ones shown in Fig. 4 b) of the main text.

Many other generalized Fano profiles were predicted and observed. In Supplementary Figure 16 a) are reported four different lineshapes calculated by Supplementary Equation (21). These profiles expand the records of the possible generalized Fano lineshapes. Noteworthy, spectra with similar behaviour can be found both by numerical FDTD calculations and by near-field experiments, as highlighted in Supplementary Figure 16 b)-c), respectively.

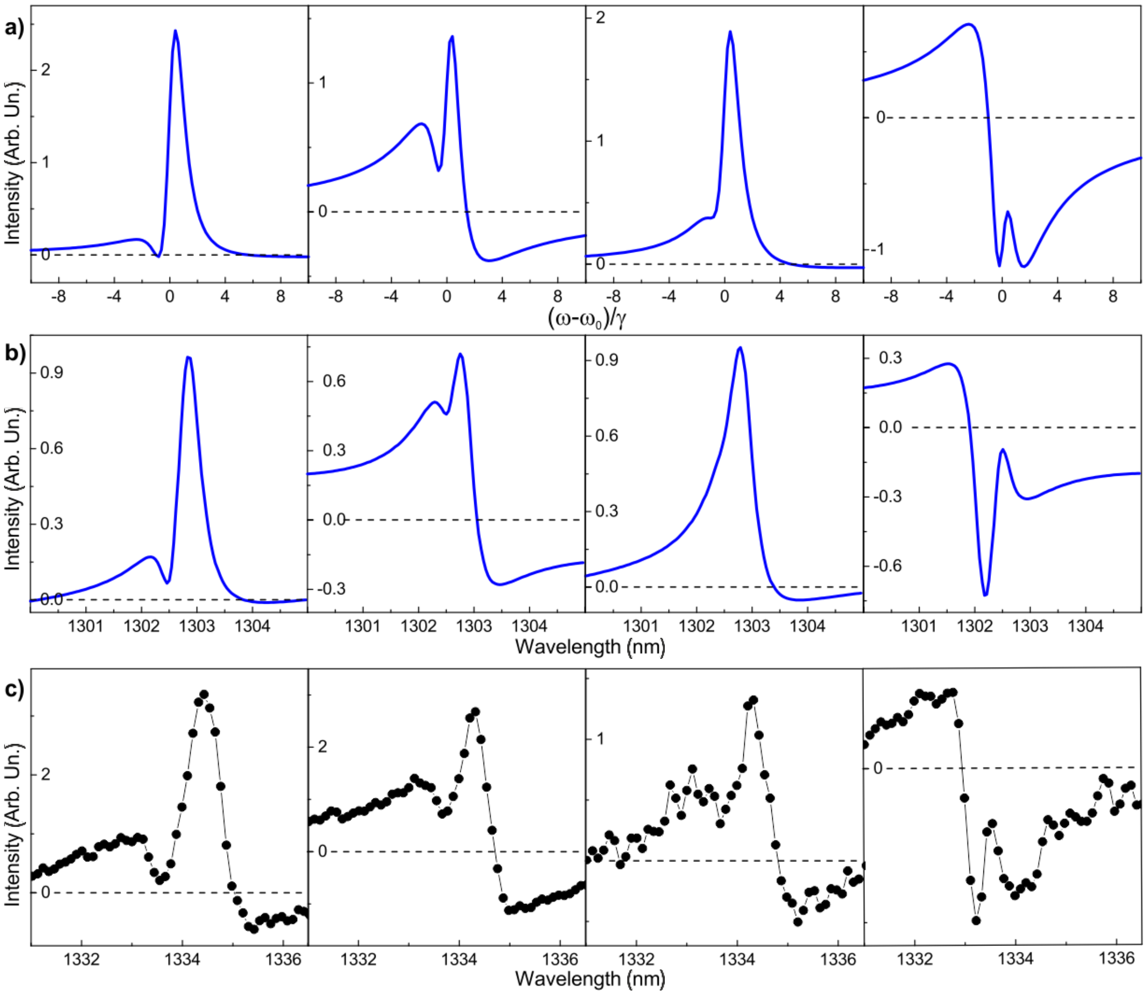

Supplementary Figure 16: Further examples of generalized Fano lineshapes. a) Spectra calculated by Supplementary Equation (21) assuming small variations between two modes: $F_B = -F_A + \delta F$ $(F_A = 1)$; $q_B = q_A + \delta q$, $\gamma_B = \gamma_A + \delta\gamma$ $(\gamma_A = 1)$ and $\omega_B = \omega_A + \delta\omega$. The used parameters are: $[\delta F = 0, q_A = 1, \delta q = -8 \cdot 10^{-4}, \delta\gamma = 10^{-3}, \delta\omega = 0]$; $[\delta F = 5 \cdot 10^{-4},\ q_A = 1,\ \delta q = -5 \cdot 10^{-4}, \delta\gamma = 10^{-3}, \delta\omega = 0]$; $[\delta F = 5 \cdot 10^{-4},\ q_A = 0.5, \delta q = 5 \cdot 10^{-4}, \delta\gamma = 10^{-3}, \delta\omega = 5 \cdot 10^{-4}]$; $[\delta F = 5 \cdot 10^{-6},\ q_A = 1, \delta q = 5 \cdot 10^{-4}, \delta\gamma = 10^{-3}, \delta\omega = 0]$, respectively. b) Near-field spectra obtained by FDTD calculations evaluated in different positions of the sample surface. c) Near-field spectra (black dots) obtained in the same scan reported in Fig. 4 b) of the main text. The connecting lines are guides to the eye.

Supplementary Note 7: Detecting small detuning

Here we discuss the possibility to detect small spectral detuning $\delta\omega$ between two modes (or two arbitrary optical signals), whenever the other parameters are well characterized. Let us consider, for example, the transmission signals from two independent resonant modes with broadening $\gamma_{\mathrm{B}} = \gamma_{\mathrm{A}} + \delta\gamma$ and frequency $\omega_{\mathrm{B}} = \omega_{\mathrm{A}}$, that result in the profile given by Supplementary Figure 3 e), calculated by Supplementary Equation (21). Now if we add a detuning $\omega_{\mathrm{B}} = \omega_{\mathrm{A}} + \delta\omega$ the lineshape changes quite dramatically, even for small variation $\delta\omega$, as reported in Supplementary Figure 7.0 a)-c) for $\delta\omega = 0$, $\delta\omega = 10^{-4}\omega_{\mathrm{A}}$, $\delta\omega = 3 \cdot 10^{-4}\omega_{\mathrm{A}}$, respectively, while $\delta\gamma = 10^{-3}\omega_{\mathrm{A}}$. This means that if the others parameters $(q_{\mathrm{A}}, q_{\mathrm{B}}, F_{\mathrm{A}}, F_{\mathrm{B}}, \delta\gamma)$ are previously known, generalized Fano lineshape allows to measure the splitting $\delta\omega$ with a quite large sensitivity.

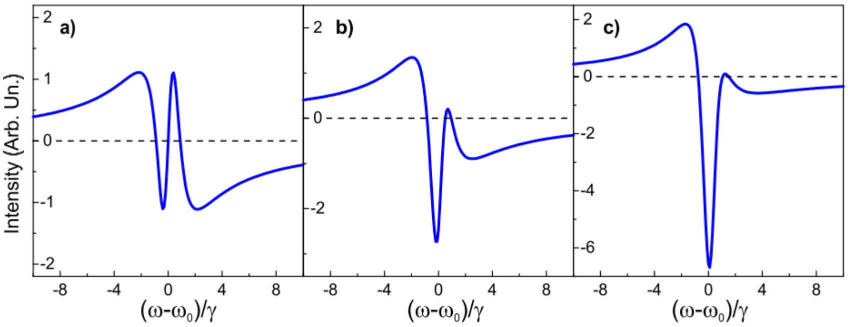

Supplementary Figure 17: Examples of generalized Fano lineshapes used for retrieving the mode detuning. We used calculated by Supplementary Equation (21) employing the parameters $q_A = q_B = F_A = F_B = 1, \gamma_B - \gamma_A = \delta\gamma = 10^{-3}\omega_A$, while the detuning was changed between a) $\delta\omega = 0$; b) $\delta\omega = 10^{-4}\omega_A$ and c) $\delta\omega = 3\text{x}10^{-4}\omega_A$.

Supplementary Note 8: Laser assisted oxidation

In order to compensate the fabrication-induced detuning between the two single cavity modes we performed a local nano-oxidation of the GaAs membrane induced by Ar-laser light (514 nm). The laser illuminates the sample through the near-field dielectric probe with a power of 2.5 mW. The non-thermal process induces a gentle blue-shift of the photonic modes [19]. Before oxidizing the sample (t=0) we mapped the mode distributions, as reported in Supplementary Figure 9.0 a)-c). At t=0 the mode with longer wavelength, labelled as P1, is localized on the right photonic cavity [see Supplementary Figure 9.0 a)]; while the shorter wavelength mode (P2) is strongly localized on the left cavity [see Supplementary Figure 9.0 b)]. This demonstrates that a disorder induced detuning overcomes the coupling between the two cavities. Then, we locally oxidized the right part of the cavity where P1 is localized [see insets of Fig. 4 a)]. Both resonant wavelengths of P1 and P2 performed a blue-shift, as highlighted in Supplementary Figure 9.0 d). The splitting variation for sequential oxidation processes gradually decreases. In fact, by exposing the Ar-laser light on the right side of the cavity system, we tune the wavelength of P1 more towards the blue than P2. Finally, at t=29 min we obtain almost degenerate modes with PL spatial distribution delocalized over the entire photonic molecule, as reported in Supplementary Figure 9.0 e)-f). By continuing the oxidation process we found that the wavelength splitting P1-P2 increases, as reported in Fig.3 a), and that the mode distributions are localized on opposite cavities; that is the larger (lower) wavelength mode P1 (P2) is found on the left (right) cavity. These features demonstrate that we performed a variation of the sign of the detuning $\delta\omega$, thus approaching the zero-value. In order to reproduce the wavelength splitting of the coupled modes, Ω =P1-P2, as a function of the oxidation time (reported in the main manuscript) we used the coupled mode theory:

$$\Omega = \sqrt{4k^2 - \delta\gamma^2 + \delta\omega^2} \tag{62}$$

Where the detuning is given by $\delta\omega = \left[A\sqrt{t - t_0} - d\right]$ as reported in [20]. The value $\delta\gamma = |\gamma_{P1} - \gamma_{P2}| = 0.05$ nm is measured at the beginning of the oxidation process when the two modes are uncoupled because of the large detuning ($\gamma_{P1} = 0.71$ nm and $\gamma_{P1} = 0.76$ nm). The fitted curve does not cross the zero value, indicating that the modes perform an anticrossing as a function of detuning and therefore they are effectively coupled. In fact, Ω is larger than zero at its minimum, where the detuning $\delta\omega$ is almost zero. The fit output parameter $t_0 = 3$ min accounts for the thin oxide layer already present when starting the oxidation process. The parameter A, which accounts for the oxidation strength, is consistent with similar oxidations reported in [20]. Finally, the coupling strength is $k = 0.03$ nm, thus resulting in a strong-coupling interaction ($2k > \delta\gamma$) between the two resonators, even if Rabi oscillation and an effective photon hopping cannot be achieved, since $k < \gamma_{P1},\ \gamma_{P2}$.

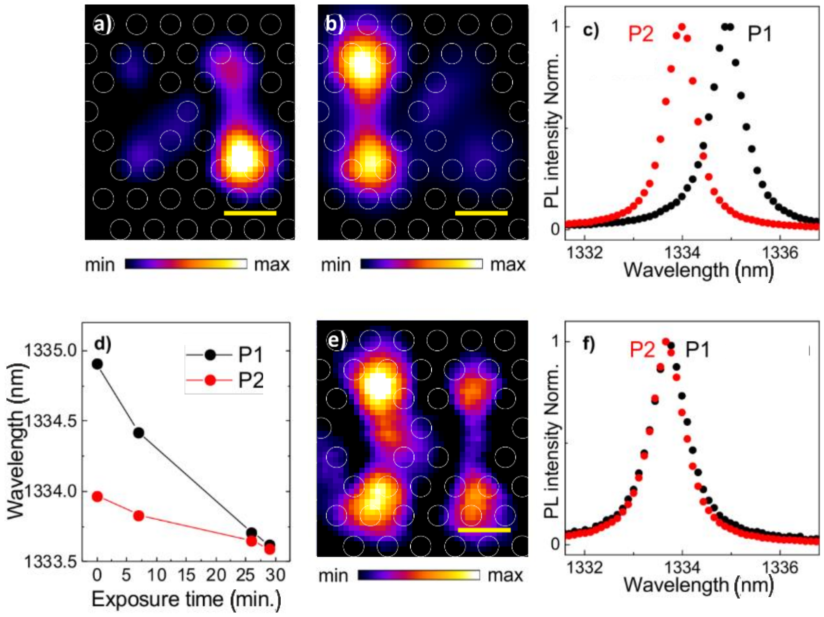

Supplementary Figure 18: Photoluminescence near-fields maps before and after the cavity nano-oxidation. a)-b) Photoluminescence maps of the modes P1 and P2 at t=0. c) Spectra on the right cavity (P1 black dots) and on the left cavity (P2 red dots) at t=0. d) Wavelength of P1 (black dots) and P2 (red dots) as a function of the laser exposure time. The connecting lines are guides to the eye. e) Photoluminescence map of the degenerate single resonance after 29 min of exposure time. f) Spectra on the right cavity (P1 black dots) and on the left cavity (P2 red dots) at t=29 min, show that the modes are almost deenerate. The scale bars are 500 nm.

Supplementary References

[1] Caselli, N. et al. Tailoring the Photon Hopping by Nearest-Neighbor and Next-Nearest-Neighbor Interaction in Photonic Arrays, *ACS Photon*. **2** (5), 565-571 (2015).

[2] Peng, B., Özdemir, Ş. K., Chen, W., Nori, F. & Yang, L. What is and what is not electromagnetically induced transparency in whispering-gallery microcavities, Nat. Commun., 5, 5082 (2014).

[3] Heiss, W. D. The physics of exceptional points, *J. Phys. A: Math. Theor.* **45** 444016 (2012).

[4] Kim, K.-H. et al. Direct observation of exceptional points in coupled photonic-crystal lasers with asymmetric optical gains, *Nat. Commun*. **7** 13893 (2016).

[5] Zhen, B. et al. Spawning rings of exceptional points out of Dirac cones, Nature, 525, 354–358 (2015).

[6] Penga, B. et al. Chiral modes and directional lasing at exceptional points, *PNAS* **113** 25 6845–6850 (2016).

[7] Houdré, R. Early stages of continuous wave experiments on cavity-polaritons, *Phys. Status Solidi B* **242**, 11 2167–2196 (2005).

[8] Brandstetter, M. et al. Reversing the pump dependence of a laser at an exceptional point, *Nat. Commun.*, **67**, 085317 (2003).

[9] Rüter, C. E. et al. Observation of parity–time symmetry in optics, *Nat. Phys.*, **6**, 3, 192-195 (2010).

[10] Ott, C. et al. Lorentz Meets Fano in Spectral Line Shapes: A Universal Phase and Its Laser Control, *Science* **340**, 6133, 716-720 (2013).

[11] Galli, M. et al. Light scattering and Fano resonances in high-Q photonic crystal nanocavities, *Appl. Phys. Lett.* **94**, 7, 1101 (2009).

[12] Totsuka, K., Kobayashi, N. & Tomita, M. Slow Light in Coupled-Resonator-Induced Transparency, *Phys. Rev. Lett.* **98**, 213904 (2007).

[13] Smith, D. D., Chang, H.K., Fuller, A., Rosenberger, A. T. & Boyd, R. W. Coupled-resonator-induced transparency, *Phys. Rev. A* **69**, 063804 (2004).

[14] Peng, B. et al. Parity–time-symmetric whispering-gallery microcavities, *Nat. Phys.* **10**, 394–398 (2014).

[15] Haus, H. A. Waves and fields in optoelectronics, *Englewood Cliffs, NJ : Prentice-Hall*, (1984).

[16] Caselli, N. *et al.* Ultra-subwavelength phase sensitive Fano-imaging of localized photonic modes, *Light Sci. and Appl.,* **4**, e326 (2015).

[17] Zhang, S., Genov, D.A., Wang, Y., Liu, M. & Zhang, X. Plasmon-Induced Transparency in Metamaterials, Phys. Rev. Lett., 101, 047401 (2008).

[18] Caselli, N., Intonti, F., Riboli, F. & Gurioli, M. Engineering the mode parity of the ground state in photonic crystal molecules, *Opt.Express*, **22**, 4953-4959 (2014).

[19] Caselli, N. et al. Post-fabrication control of evanescent tunnelling in photonic crystal molecules, *Appl.Phys.Lett.*, **101**, 211108 (2012).

[20] Intonti, F. et al. Mode tuning of photonic crystal nanocavities by photoinduced non-thermal oxidation, *Appl. Phys. Lett.* **100**, 033116 (2012).
